# Integrative and conjugative elements in *Mycoplasmopsis bovis* from Western Canadian feedlot cattle: characterization and conjugative transfer

**DOI:** 10.3389/fvets.2026.1719776

**Published:** 2026-01-27

**Authors:** Sara Andres-Lasheras, Rahat Zaheer, Rodrigo Ortega-Polo, Timothy Schwinghamer, Sujeema Abeysekara, Athanasios Zovoilis, Sani-e-Zehra Zaidi, Murray Jelinski, Tim A. McAllister

**Affiliations:** 1 Lethbridge Research and Development Centre, Agriculture and Agri-Food Canada, Lethbridge, AB, Canada; 2 Department of Biochemistry and Medical Genetics, University of Manitoba, Winnipeg, MB, Canada; 3 Western College of Veterinary Medicine, University of Saskatchewan, Saskatoon, SK, Canada

**Keywords:** antimicrobial resistance, epidemiology, horizontal gene transfer, integrative and conjugative elements, *Mycoplasmopsis bovis*

## Abstract

**Introduction:**

Bovine respiratory disease (BRD) is the most significant disease affecting North American feedlot cattle. It is a multifactorial disease influenced by bacterial and viral pathogens, as well as management and environmental factors. *Mycoplasmopsis bovis* is among the most pathogenic bovine mycoplasmas and is associated with chronic BRD that often fails to respond to antimicrobial therapy. Integrative and conjugative elements (ICE) facilitate horizontal gene transfer among mycoplasmas and may contribute to the spread of antimicrobial resistance in *M. bovis*.

**Methods:**

We identified mycoplasma ICEs (MICE) in the genomes of sequenced *M. bovis* isolates from western Canadian feedlot cattle (*n* = 124) and in vitro mating experiments to assess conjugation.

**Results and Discussion:**

Of these isolates, 33.1% harbored the array of MICE genes required for conjugation. *M. bovis* isolates conjugated at frequencies of 10–7–10–8 when cultured in SP4 broth under orbital agitation. Since MICE circularization is the initial step in conjugation, the presence of circular MICE (cMICE) was used as a proxy for conjugation capability (*n* = 451). Interestingly, 25.7% of the isolates were cMICE-positive, with a higher prevalence observed in *M. bovis* isolated from dairy as compared to beef feedlot cattle. Additionally, calves classified as high-risk for BRD were more likely to harbor cMICE-positive *M. bovis* in both cattle types. Backgrounded dairy cattle had a higher likelihood of carrying cMICE-positive *M. bovis* than those originating from ranches. These findings lay the groundwork for assessing cattle source as a determinant of cMICE-positive *M. bovis* and for developing targeted strategies to mitigate antimicrobial resistance.

## 1 Introduction

Bovine respiratory disease (BRD) is the most significant health and economic challenge affecting feedlot cattle in North America (1). Together with *Mannheimia haemolytica*, *Pasteurella multocida*, and *Histophilus somni*, *Mycoplasmopsis bovis* is one of the main opportunistic bacteria involved in BRD (2–4). Among mycoplasma species, *M. bovis* is one of the most pathogenic and is associated with chronic pneumonia, polyarthritis syndrome, mastitis, genital disorders, otitis media, and keratoconjunctivitis (5). Control of *M. bovis* infections is hampered by a lack of effective vaccines and increasing resistance to antimicrobials in Europe and North America (6–9).

Mobile genetic elements (MGE), such as plasmids, transposons, and integrative and conjugative elements (ICE) are known to play a key role in the horizontal dissemination and persistence of antimicrobial resistance (AMR) in bacteria (10). Integrative and conjugative elements are MGEs that integrate into the bacterial host chromosome with the capacity for self-excision from the donor cell, circularization, horizontal transmission by conjugation to a recipient cell, and integration into the recipient cell chromosome (11). These MGEs have been recognized as the most abundant conjugative elements in prokaryotes, outnumbering conjugative plasmids (12). Despite their reduced genomes (13), mycoplasma species exhibit remarkable genomic plasticity, largely driven by ICE-mediated horizontal gene transfer. A comprehensive analysis of 1,433 *Mollicutes* genomes (including *Mycoplasma*, *Ureaplasma*, and *Spiroplasma*) revealed that 83.9% of these species show evidence of horizontal gene transfer (HGT), with ICEs and integrative and mobilizable elements (IMEs) playing a central role (14). ICEs are capable of cross-species transfer, particularly among *Mycoplasma* species sharing ecological niches, and play a pivotal role in the adaptive evolution of these pathogens through creating mosaic genomes (14). Integrative and conjugative elements are prevalent in *M. bovis* (15, 16), but unlike ICE in BRD-associated *Pasteurellaceae*, mycoplasma ICEs (MICE) do not carry AMR genes (13, 17). However, MICEs have been associated with the horizontal transfer of virtually any gene between mycoplasma cells, including those associated with AMR by point mutations (18). *Mycoplasmopsis agalactiae* has been used extensively as a conjugation model, and HGT has been documented among *M. agalactiae* strains, between *M. agalactiae* and *M. bovis*, and among *M. bovis* strains (18, 19). Horizontal, MICE-mediated transfer of enrofloxacin-resistance has been demonstrated between *M. agalactiae* cells (20), but the horizontal transmission of AMR across *M. bovis* and other mycoplasma species relevant in feedlot cattle has not been a subject of previous research.

Building on existing evidence that MICEs mediate horizontal gene transfer in mycoplasma species, we hypothesized that North American *M. bovis* possesses functional MICE capable of conjugation, potentially facilitating the transfer of genes with mutations that contribute to AMR. Therefore, the objectives of this study were to document the presence of MICE and epidemiology of circular MICE (cMICE) in *M. bovis* isolated from feedlot cattle in western Canada; to characterize the structure and diversity of the detected MICE; to perform a comparative genomic analysis of such MICE; and to determine the *in vitro* conjugation capabilities of *M. bovis* through MICE.

## 2 Materials and methods

### 2.1 Isolates and culture

Field isolates of *M. bovis* were sourced from two different collections originating in western Canada (TMC and MJC collections) (Supplementary material 2, Table S1). The TMC collection included 455 respiratory isolates (used for cMICE epidemiological studies), each from a different calf, sampled from 10 different feedlots between 2017 and 2019 (9). Isolates in the MJC collection (*n* = 211; used for MICE genomic analyses) were obtained from 96 feedlot cattle from different types of samples, across 21 different feedlots, and between 2006 and 2018 (6). For conjugation studies, *M. agalactiae* reference strain PG2 (NCTC 10123) was kindly provided by Dr. John Devenish (CFIA, Canada).

All *M. bovis isolates*, transformants, and transconjugants were grown at 37 °C in SP4 broth (Supplementary material 2, Table S2), with or without 5% CO_2_ according to experimental conditions (21), and without ampicillin unless otherwise specified. Additional media formulations, including N broth, PPLO, Eaton’s, and SP4 with fetal bovine serum (FBS) substituted by Proliferum (Multus Biotechnology; London, UK), were tested for their suitability for *M. bovis* conjugation studies (Supplementary material 2, Tables S3, S4). For culturing transformants and transconjugants, SP4 medium with puromycin (PURO; 5 μg/mL), gentamicin (GEN; 50 μg/mL), or tetracycline (TET; 8 μg/mL), either individually or in combination, was used (22). The concentration of tetracycline in SP4 media for the growth of TFs was 4-fold higher than the previously recommended 2 μg/mL (23) due to the widespread of tetracycline resistance among *M. bovis* isolates (6, 9). At 2 μg/mL (22), tetracycline was found to not inhibit the growth of *M. bovis*. Therefore, the maximum tetracycline concentration tolerated by *M. bovis* TET-transformants was determined using SP4 agar (data not shown).

### 2.2 Bacterial genome sequencing and assembly

For the study of MICE and the identification of conjugation candidates, we leveraged 120 genomes previously sequenced from the MJC collection of *M. bovis* using Illumina short read technology (BioProject ID PRJNA642970; Supplementary material 3, Tables S5, S6) (24). Briefly, genomic library construction was performed using the Illumina Nextera XT DNA sample preparation kit (Illumina Inc.; San Diego, CA, USA). Libraries were sequenced on an Illumina MiSeq platform using the MiSeq Reagent Kit V2 to generate 2 × 250 bp paired-end reads. Additionally, several isolates from the TMC and MJC collections were subjected to Illumina short read (re)sequencing to support hybrid genomic assemblies with long read sequencing and/ or for MICE characterization (Supplementary material 3, Tables S5, S7). Specifically, isolates with relevant antimicrobial susceptibility profiles (6, 9), MICE types (*Genomic analyses* section), or cMICE presence (*cMICE epidemiology* section) were sequenced, i.e., *M. bovis* from MJC [646, 645, 643, 019; *n* = 124 in total] and TMC [J288, C176, J10]. For this, the aforementioned short read sequencing procedure was used with the only difference being that the MiSeq Reagent Kit V3 was used to generate 2 × 300 bp paired-end reads. For long read sequencing (*n* = 10; Supplementary material 3, Tables S7–S9), high molecular weight genomic DNA was extracted using the Qiagen HMW Blood & Cell Culture DNA Kit with Genomic-tip 20/G (Qiagen, Toronto, ON, Canada). DNA was end-repaired using the NEBNext Ultra II End Repair/dA-Tailing kit (New England Biolabs Ltd. Whitby, ON, Canada). Barcoding was performed using the Oxford Nanopore barcoding kit EXP-NBD196), and samples were pooled and cleaned to remove reagents of the previous step(s) using the Omega-bind NGS beads (Omegabiotek, M1378-01) following manufacturer’s instructions. Sequencing adapters were then ligated using Adapter Mix II Expansion kit EXP-AMII001 (Nanopore Technologies) and sequenced on a PromethION sequencing platform. MinKNOW Core 3.1.20 and guppy 2.0.10 were used for flow cell signal processing and base calling in real time.

The Galaxy platform (25) was used to check the quality and processing of Illumina raw reads. Read quality was assessed using FastQC v0.12.1 (26), and trimming was performed with Trimmomatic (Galaxy v0.39 + galaxy2) using the following parameters: head crop = 19, trailing = 20, and minimum length = 36 bp (27). Draft genome assemblies were generated *de-novo* using Illumina short-reads only (Shovill pipeline v1.1.0) (28), and annotation was performed with Prokka (Galaxy v1.14.6 + galaxy1) (29) using the *M. bovis* PG45 genome as reference (NC_014760.1). For hybrid assembly of *M. bovis* genomes, the *Mycovista* pipeline (30) was used as it specifically addresses highly repetitive genomes like *M. bovis*. *Mycovista* used Oxford Nanopore long reads as the reference assembly in combination with Illumina short reads for polishing. The pipeline was run in a high-performance computing cluster, and it was modified to use Apptainers (i.e., containers) instead of Conda environments. Apptainers with the same versions of tools as in the published Mycovista workflow were used. All default hybrid assembly pipeline parameters were retained with the exception of Trimmomatic which was set with the same trimming settings as described above for Illumina short reads pre-processing. Hybrid genomes were quality checked using Quast (included in *Mycovista*; v5.0.2) and BUSCO (v5.8.0; *Metaeuk* gene predictor, *Mycoplasmatales* Lineage).

### 2.3 Genomic analyses

*Mycoplasmopsis bovis* MICE genomic analyses were carried out using published sequences as a reference for hominis-type (H-type) MICE which included *Mycoplasmopsis agalactiae* 5,632 ICEA_5632_-I (GenBank: CT030003.1) and *M. bovis* PG45 ICEB-2_PG45_ (NCBI accession number: NC_014760.1); and spiroplasma-type (S-type) MICE *M. bovis* PG45 vICEB-1_PG45_ (vICE, vestigial ICE; NCBI accession number: NC_014760.1) (16). Coding regions (CDS) CDS1, 13, 14, 15, 16, 17, 19, 22, 30, 5, 7, A, and G in ICEB-2_PG45_ [CDSs identified in the literature as essential for conjugation in *M. agalactiae* strain 5,632 (22)] and CDS3, 14, 15, 16, 17, 19, 22 in vICEB-1_PG45_ were searched (minimum alignment coverage threshold of 70% and a sequence identity cutoff of 90%). The CDSC is not annotated in ICEB-2_PG45_, but it is described as part of the MICE conjugative machinery in ICEA_5632_-I (22) and was therefore included in the screening (minimum alignment coverage threshold of 35% and a sequence identity cutoff of 50%). Additionally, the two non-coding regions (ncr) *ncr16-27* and *ncrD-5* were extracted because of their potential role in conjugation as regulatory and/ or *cis*-acting elements (22). All MICE-CDS screening were carried out in Geneious (v.10.2.6) using the custom BLAST tool to determine presence/absence.

*Mycoplasmopsis bovis* draft genomes from MJC collection (*n* = 124; Supplementary material 3, Table S5) were screened for the presence/absence of the aforementioned MICE-CDSs and ncr, whereas hybrid assemblies were used for a more detailed comparative study of the structure and composition of H and S-type MICE. A total of 41 hybrid assemblies were included for the analyses, i.e., 10 generated in this study and 31 from the literature (30–32) (Supplementary material 4, Table S10). MICE from hybrid assemblies were manually annotated as specified elsewhere (16). Vestigial MICE that contained a few MICE-CDSs were excluded from the hybrid assembly studies, i.e., for H-type MICE, only those harboring the CDSs from CS1 to CDS22 were included; likewise, for S-type MICE, CDS3 to CDS22 were included. Additionally, 12 *M. agalactiae* genomes (Accession numbers: GCA_009150585.1, GCA_012689495.1, GCA_019552405.1, GCA_024582795.1, GCA_036542405.1, GCA_036542425.1, GCA_036542445.1, GCA_036542465.1, GCA_036549555.1, GCA_900088695.1), at the complete or scaffold level, were retrieved from the NCBI database (May 2025) and searched for H and S-type MICE by BLAST as specified for *M. bovis*.

### 2.4 Circular MICE: qPCR assay development and culture conditions

Initial cMICE screening, performed using previously described cPCR with left1/right2 primers (16), identified *M. bovis* 646 as cMICE-positive. The resulting *M. bovis* 646 cMICE cPCR amplicon was verified by Sanger sequencing (Eurofins Genomics) (16) and used to develop a qPCR assay that targeted the cMICE junction region (primers designed in Geneious v10.2.6; cMICE-F: 5′ – TCTTATGCATAGAAGTAAAGTAGAGT – 3′; cMICE-R: 5′ – ACCCACTTTCTTCTATCAGTTC – 3′) (Supplementary materials 5, 6). Subsequently, different culture/environmental conditions in SP4 broth were explored, individually or in combination, to identify those associated with the largest quantity of cMICE, i.e., growth at different phases, temperatures, cell densities, pH, nutrients availability, UV light exposure, mitomycin C exposure, and atmospheric CO_2_ concentrations (Supplementary material 7). Standard culture conditions were set at 37 °C, 5% CO_2_, without agitation. Each treatment was tested in 3 independent experiments (biological replicates, BR). For each environmental stressor, mycoplasma cell survival was assessed before and after treatment by *M. bovis* colony enumerations on SP4 agar. The gDNA of treated and un-treated, control cultures was extracted using the *DNeasy Blood & Tissue* DNA isolation kit (Qiagen). From each treated and control sample, the relative cMICE quantity was determined by the ΔΔCt method in a *StepOnePlus* qPCR thermocycler (Applied Biosystems). The single-copy, housekeeping *uvrC* gene was used as the endogenous standard (33) and SYBR green chemistry was used for fluorescence measurements. The ΔΔCt method followed Pfaff’s principle (34) which allows for relative quantification of a target compared to a reference gene, enabling comparison between treated and control samples without the need of calibration curves (each BR was qPCR-tested in triplicates). cMICE quantity levels were compared across treatments using the Kruskal-Wallis test (*p* < 0.05).

### 2.5 cMICE epidemiology

The presence of cMICE was used as a proxy of conjugation capabilities amongs 451 *M. bovis* isolates from the TMC collection (9). This collection was obtained from a previous cross-sectional study aiming to determine AMR levels at feedlot entry of the 4 main bacterial species involved in BRD, including *M. bovis*. For that study, deep nasopharyngeal swabs (DNPS, *n* = 2,824) were collected during two sampling periods (August 2017–May 2018 and August 2018–April 2019) from 10 different feedlots in Alberta, Canada. From each calf, only one DNPS was obtained before antimicrobials were administrated at processing, and a series of epidemiological factors were recorded to investigate their possible relationship with higher AMR sources, i.e., arrival date, cattle type (beef, dairy), sex (heifer, bull, steer), weight (kg), age class (calf, yearling), origin (ranch direct, auction barn, backgrounding operation), feedlot, and BRD risk during the feeding period (high, low) (9). Transport trailer was considered the primary sampling unit, with cattle from the same truckload considered a cluster (STC or same truck load). The majority of beef-type cattle were sourced through auction marts whereas most dairy-type cattle were farm-direct. When auction mart beef cattle were from different locations, but transported in the same truck, the random effect was nested (arrived from within STC). For dairy-type cattle models, 2 random effects were included (STC and arrived from) when the location of their origin was known. cMICE screening was carried out for each *M. bovis* isolate following the above described direct-qPCR assay.

*Mycoplasmopsis bovis* isolates were grown in SP4 broth (1 replicate/isolate), without shaking, to early stationary phase. Growth phase was confirmed by broth turbidity either manually (spectrophotometer Genesis 20, Thermo Scientific) or automatically (Stratus, Cerillo, Charlottesville, VA) [absorbance at 450 nm (35)]. To validate results, a subset of direct-qPCR-screened cMICE positive (*n* = 35) or negative (*n* = 22) *M. bovis* were subjected to gDNA extraction followed by the detection of cMICE by conventional PCR (cPCR) using the published left1/right2 primers (Supplementary material 6) (23). To further verify that the binding sites of the new cMICE qPCR primers were conserved across isolates, a subset of cPCR amplicon products (*n* = 25/35) were Sanger sequenced (Eurofins Genomics) and analyzed *in silico* using the Geneious Alignment Tool (Geneious v10.2.6).

Generalized linear mixed models (GLMM; GLIMMIX procedure in SAS) were used to model cMICE as a binary response variable (presence/absence). Age, country, feedlot, risk, season, sex, source, weight, and year were initially included as fixed factors and dropped from the models where not statistically significant. Feedlot capacity was not included in the models owing to the relatively problematic level of multicollinearity, as quantified by the variance inflation factor (data not shown). The proposed random effects were dropped from the models when the GLIMMIX procedure did not converge. Models were run for the full dataset and also for data stratified by cattle type since the relevancy of this variable was confirmed in previous AMR studies (9). For beef-type models, the samples from the US were excluded because none of the samples were cMICE-positive (*n* = 0/5). Likewise, in the dairy-type dataset, samples from auction (*n* = 0/1 cMICE positive) and female (*n* = 0/2 cMICE positive) cattle were excluded.

### 2.6 Bacterial transformation

*Mycoplasmopsis bovis* transformation was carried out as previously described (23) using the pMT85 derived plasmids to tag isolates with antimicrobial resistance genes (ARG) conferring resistance to PURO, GEN, or TET (22). The pMT85 plasmids carry a mini transposon (Tn) composed of the ARG cassette flanked by two Tn4001 inverted repeats (IR). The mini-Tn4001 lacks the transposase gene, resulting in the stable insertion of the ARGs in the mycoplasma chromosome that prevents spontaneous relocation of the resistance gene markers within the mycoplasma chromosome.

The insertion of ARGs in the *M. bovis* genome was verified using a direct qPCR approach aiming to bypass genomic DNA (gDNA) extraction (33). For this, new qPCR assays were developed to detect PURO, GEN, and TET genes (primers designed in Geneious v.10.2.6) (Supplementary materials 5, 6). The performance of these assays was validated by amplifying the corresponding ARGs from purified gDNA of mycoplasma transformants and purified plasmid DNA (pDNA). For this, nine *M. bovis* 057 PURO transformants, nine *M. bovis* 643 GEN transformants, and nine *M. bovis* D317A TET transformants were subjected to gDNA extraction using the *DNeasy Blood & Tissue* DNA isolation kit (Qiagen), followed by cPCR using primers Gm1/Gm2, IntMtet1/IntMtet2, and purF/purR for the amplification of GEN, TET, and PURO genes, respectively (Supplementary material 6) (23).

### 2.7 Conjugation studies

Conjugation in *M. bovis* was assessed using a previously developed reference method (21). To verify conjugates, *M. bovis* transformants carrying pMT85-PURO and pMT85-GEN plasmids (transformants designated as *Mbov*^P^ and *Mbov*^G^, respectively) were used as mating parents (23). Unless otherwise stated, conjugation parents consisted of pools of either Mbov^P^ or Mbov^G^ transformants (up to 9 different transformants/pool), instead of individual transformant colonies to increase the likelihood of detecting conjugation events (23). Initially, conjugation was attempted using *M. bovis* 646 as one of the parenteral isolates since it was the first one identified as cMICE positive (Supplementary material 8, Table S14). Due to the absence of conjugation mating *M. bovis* 646 with *M. agalactiae* PG2, conjugation was attempted in a series of 20 mating experiments involving 10 different *M. bovis* field isolates and *M. agalactiae* PG2, in different combinations (Supplementary material 8, Table S14). However, successful conjugation was observed in only 2 out of 20 experiments. In both cases, the same parent containing ICEB-2, *M. bovis* isolate 643, successfully conjugated with either *M. bovis* isolate I100 or 057. Therefore, to optimize conjugation efficiency, various experimental parameters were tested, including incubation time, parent-to-parent ratios, media type, and agitation, to determine their impact on MICE-mediated conjugation in *M. bovis* (Supplementary material 9). Among these, orbital agitation was the only factor that consistently increased conjugation between *M. bovis* 643 and I100 (Supplementary material 8, Table S15). As a result, agitation was incorporated into the optimized conjugation protocol for *M. bovis* in SP4 broth (Supplementary material 10). Three independent conjugation experiments using *M. bovis* 643^G^ x I100^P^ were conducted to determine conjugation frequency and optimal incubation time. After cloning and filtering (21), all suspected transconjugants were screened using direct qPCR for the presence of both PURO and GEN markers. Transconjugants testing positive for both markers were further verified by gDNA purification and cPCR using previously published primers (Supplementary material 6).

## 3 Results

### 3.1 Isolates and culture

SP4 media has been used for mycoplasma conjugation experiments before (21), but it can be expensive due to the supplements it contains such as FBS. Consequently, different media were tested for their suitability in this study (Supplementary material 2). SP4 outperformed the other media formulations tested (N, PPLO, and Eaton’s; Supplementary material 2, Table S3), and the substitution of FBS by Proliferum did not support optimum growth of *M. bovis* in SP4 (Supplementary material 2, Table S4, Figures S1, S2).

### 3.2 Bacterial genome sequencing and assembly

Draft genomes of the 124 MJC isolates produced assemblies with an average N50 of 21,391 bp (range: 1,952 to 45,260 bp) and an average of 212 contigs per genome (range: 123 to 488) (Supplementary material 3, Table S6). The 10 hybrid assemblies presented from 1 to 4 contigs with a largest contig length range of 976,369–1,154,934 bp and a CG average content of 29.2% (Supplementary material 3, Table S8). In 2 instances, 1 and 2 genes (BUSCOs) were missing in a total of 4 hybrid assemblies out of a total of 174 BUSCOs included in the quality analysis (Supplementary material 3, Table S9).

### 3.3 Genomic analyses

The draft genomes obtained from MJC isolates were screened for the presence/absence of hominis and spiroplasma-type MICE-CDSs. All the draft genomes included (*n* = 124) contained at least one MICE-CDS (sequence identity range 94.8–100%), with 64.5% (80/124) presenting MICE-CDSs of two MICE types, i.e., hominis and spiroplasma (Tables 1, 2). Spiroplasma-like MICE-CDSs were found in all genomes, either alone or in consort with hominis-like MICE-CDSs in the same genome, whereas hominis-like MICE-CDSs were only found as genome co-residents of the spiroplasma-type. Half (41/80) of the isolates with hominis-type MICE-CDSs presented all the MICE-CDSs identified in the literature as essential for conjugation in *M. agalactiae* strain 5,632 (Table 1) (22), meaning that 33.1% (41/124) of the isolates (which included *M bovis* 643) presented potentially functional hominis MICE.

**Table 1:** Hominis-type MICE-CDSs detected using ICEB-2_PG45_ as a reference (*n* = 80/124).

MICE-CDS profile	Number of isolates	%
CDS1	14	17.5
CDS13-CDS14-CDS15-CDS16-CDS17-CDS19-CDS22-CDS30-CDS5-CDS7-CDSA-CDSG	3	3.8
CDS13-CDS14-CDS15-CDS16-CDS17-CDS19-CDS22-CDS30-CDSA-CDSG	1	1.3
CDS13-CDS14-CDS15-CDS16-CDS19-CDS22-CDS30-CDS5-CDS7-CDSA-CDSG	1	1.3
CDS13-CDS14-CDS15-CDS16-CDS19-CDS22-CDS5-CDS7-CDSA-CDSG	1	1.3
CDS13-CDS14-CDS15-CDS16-CDS22-CDS30-CDS5-CDS7-CDSA-CDSG	1	1.3
CDS13-CDS15-CDS16-CDS17-CDS19-CDS22-CDS30-CDS5-CDSG	1	1.3
CDS13-CDS15-CDS16-CDS22-CDS30-CDS5-CDS7-CDSA-CDSG	1	1.3
CDS13-CDS15-CDS22-CDS7	1	1.3
CDS1-CDS13-CDS14-CDS15-CDS16-CDS17-CDS19-CDS22-CDS30-CDS5-CDS7-CDSA-CDSG	41	51.3
CDS1-CDS13-CDS14-CDS15-CDS16-CDS19-CDS22-CDS30-CDS5-CDS7-CDSA-CDSG	10	12.5
CDS1-CDS13-CDS14-CDS15-CDS16-CDS19-CDS22-CDS30-CDS5-CDS7-CDSG	1	1.25
CDS1-CDS13-CDS15-CDS16-CDS17-CDS19-CDS22-CDS30-CDS5-CDSA-CDSG	2	2.5
CDS1-CDS13-CDS15-CDS16-CDS19-CDS22-CDS5-CDS7-CDSA-CDSG	1	1.3
CDS1-CDS13-CDS15-CDS16-CDS22-CDS30-CDS5-CDS7-CDSA-CDSG	1	1.3

CDS, coding sequence; MICE, mycoplasma integrative and conjugative element. The CDS order presented in this table does not represent CDS synteny within a MICE, it only represents presence.

**Table 2:** Spiroplasma-type MICE-CDSs detected using ICEB-1_PG45_ as a reference (*n* = 124/124).

MICE-CDS profile	Number of isolates	%
CDS14-CDS15-CDS16-CDS22-CDS3	26	21.0
CDS14-CDS15-CDS16-CDS17-CDS22-CDS3	27	21.8
CDS14-CDS15-CDS16-CDS17-CDS3	4	3.2
CDS14-CDS15-CDS16-CDS17-CDS19-CDS22-CDS3	24	19.4
CDS14-CDS15-CDS16-CDS17-CDS19-CDS22	1	0.8
CDS14-CDS15-CDS16-CDS19-CDS22-CDS3	6	4.8
CDS14-CDS15-CDS16-CDS3	7	5.6
CDS14-CDS15-CDS16	1	0.8
CDS14-CDS15-CDS16-CDS17-CDS19-CDS3	3	2.4
CDS14-CDS15-CDS16-CDS17-CDS22	2	1.6
CDS14-CDS15-CDS16-CDS19-CDS3	1	0.8
CDS15-CDS16-CDS22-CDS3	2	1.6
CDS15-CDS16-CDS3	4	3.2
CDS15-CDS16	3	2.4
CDS22-CDS3	13	10.5

CDS, coding sequence; MICE, mycoplasma integrative and conjugative element. The CDS order presented in this table does not represent CDS synteny within a MICE, it only represents presence.

Compared to ICEA_5632_-I, *M. bovis* H-type MICEs from all the draft and hybrid assemblies lacked CDSH. The CDS13 from *M. bovis* ICEB-2_PG45_ had to be manually annotated for every *M. bovis* H-type MICE. The CDSC was detected in H-type *M. bovis* MICE with a 58.4–62.6% of pairwise identity (size range: 211–298 bp; CDSC from ICEA_5632_-I is 561 bp) and aligned with CDS11 from ICEB-2_PG45_ (MBOVPG45_207 and MBOVPG45_208, of 660 and 636 bp, respectively) and vICEB-1_PG45_ (MBOVPG45_489 and MBOVPG45_488, of 663 and 660 bp, respectively). The *ncr16-27* presented an 88.3–89.7% pairwise identity (146 bp in all cases, matching the size in ICEA_5632_-I), whereas the *ncrD-5* exhibited lower identity (46.9–52.9%) with 50–51 bp length, except for one instance of 64 bp (compared to 94 bp in ICEA_5632_-I). Notably, 17.5% of draft (*n* = 14/80 MJC isolates presenting H-type MICE) and 36% of hybrid (*n* = 9/25 from TMC and NCBI) *M. bovis* assemblies contained CDS1 alone from H-type MICE.

Mycoplasma hybrid assemblies were used for a comparative analysis of MICE structure. The overall structure and synteny of *M. bovis* MICE were conserved across isolates (Supplementary material 4, Figures S3, S4). Insertion sequences (IS) were present within all the studied H-type and in most of S-type MICE (29/38) in *M. bovis*, but were absent in *M. agalactiae* MICE. In *M. bovis*, IS were found both between and within MICE-CDSs. Out of the 12 *M. agalactiae* genomes (including strains PG2 and 5,632), only 2 strains, 5,632 and 4,867 harbored H-type MICE (Supplementary material 4, Figure S3), whereas 8 out of 12 had S-type MICE.

### 3.4 Circular MICE: qPCR assay development and culture conditions

The presence of cMICE was used as a proxy of conjugation capabilities on *M. bovis* isolates from the TMC collection. For that, a new qPCR assay was developed targeting the cMICE junction region. *In silico* sequence alignments showed that the qPCR primer binding sites were conserved across all sequenced isolates (data not shown). The direct-qPCR limit of detection (LOD) showed a positive result for bacterial growth in SP4 broth at concentrations between 10^9^ and 10^7^ CFU/mL (Supplementary material 5). Among the 57 cMICE PCR assays performed, 56 (98.3%) showed a concordant result between cPCR and qPCR, with only one sample being qPCR-positive but cPCR-negative (Table 3).

**Table 3:** Comparative results of the screening of cMICE by qPCR and cPCR.

Number of isolates	cPCR: gDNA + published primers	Direct qPCR: 2 μL broth culture + new primers
34	+	+
22	−	−
1	−	+

cMICE, circular mycoplasma integrative and conjugative element; cPCR, conventional PCR; gDNA, genomic DNA; qPCR, quantitative PCR.

**Figure 1:**
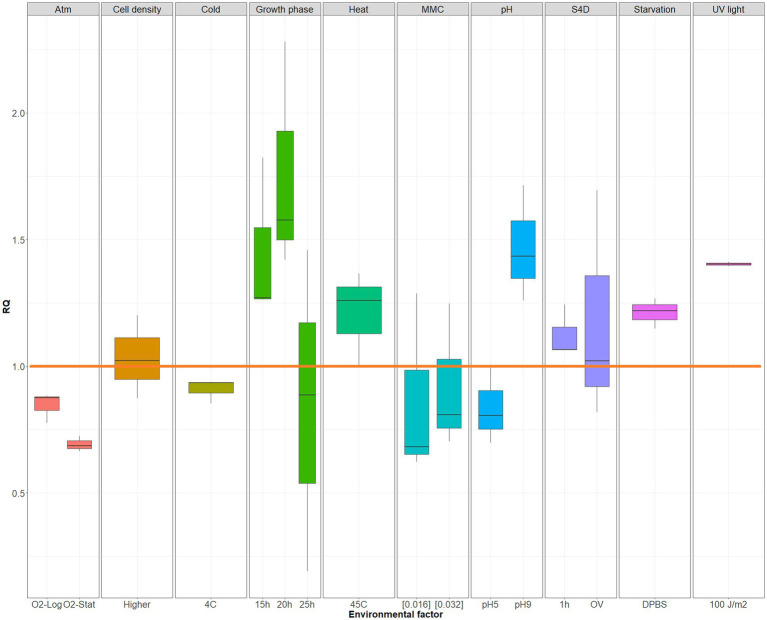
Relative expression ratio (RQ, linear scale) of cMICE under different culture conditions/stressors. RQ values per environmental factor: representation of three biological replicates (each containing 3 technical replicates). Orange, horizontal line represents the RQ value for controls, i.e., the RQ baseline value in a linear scale (equal to 1); cMICE, circular MICE. Atmosphere in logarithmic phase (Atm-Log) *p* = 0.9999; atmosphere in stationary phase (Atm-Stat) *p* = 0.9932; cold (4 °C) *p* = 1; higher *M. bovis* cell density *p* = 1; growth at 15 h *p* = 0.8456, 20 h *p* = 0.1318, and 25 h *p* = 0.9999; heat (45 °C) *p* = 0.9999; mitomycin C (MMC; sub-inhibitory concentrations tested at μg/mL) [0.016] *p* = 1, and [0.032] *p* = 1; pH equal to 5 *p* = 0.9999, and 9 *p* = 0.8075; starvation (DPBS) *p* = 0.9999; starvation (DPBS) + cold (4 °C) + higher *M. bovis* cell density (S4D) followed by an incubation under standard conditions of 1 h *p* = 1, or overnight (OV) *p* = 0.9999; UV light (100 J/m^2^) *p* = 0.9705.

Once the cMICE qPCR assay was developed, different environmental factors were tested to determine if they had an effect on cMICE quantity. Although some of the environmental conditions showed varying cMICE quantity ratios (Figure 1), none differed significantly from the non-treated control (*p* > 0.05). However, cells at early stationary phase (20 h) possessed the highest cMICE (RQ value >2). Due to the simplicity of culturing *M. bovis* isolates to the early stationary phase in SP4 broth, this approach was selected to study cMICE epidemiology. Culture conditions assessed for the quantification of cMICE did not impact the viability of *M. bovis* 646, as confirmed by colony enumeration (Supplementary material 7).

### 3.5 cMICE epidemiology

To estimate the conjugation capabilities of the entire TMC *M. bovis* isolates collection (*n* = 451; 4/455 isolates did not grow when retrieved from long term storage), the new cMICE qPCR assay was used for the isolates growing under those environmental conditions previously determined to have higher cMICE quantities. Of the 451 *M. bovis* isolates tested, 25.7% (*n* = 116) were cMICE-positive (Table 4). The prevalence of cMICE varied by cattle type, with 41.3% of isolates from dairy cattle testing positive, compared to 13.7% from beef cattle (Table 4).

**Table 4:** Proportion of positive *M. bovis* cMICE in total samples and as stratified by cattle type from various sources.

Risk factor	Risk factor levels	Total samples *n* = 116/451 (25.7%)		Beef type *n* = 35/255 (13.7%)		Dairy type *n* = 81/196 (41.3%)	
		*n*	%	*n*	%	*n*	%
Country	Canada	71/307	23.1	35/250	14.0	36/57	63.1
	USA	45/144	31.5	0/5	0.0	45/139	32.4
Source	Ranch direct	90/272	33.1	15/89	16.9	75/183	41.08
	Backgrounding	19/89	21.4	13/77	16.9	6/12	50.0
	Auction	7/90	7.8	7/89	7.9	0/1	0.0
Sex	Female	12/88	13.7	12/86	14.0	0/2	0.0
	Male	104/363	41.3	23/169	13.6	81/194	41.8
Age	Calf	97/296	13.6	21/112	18.8	76/183	41.5
	Yearling	19/155	28.7	14/143	9.8	5/11	45.5
BRD risk	High	89/231	32.8	19/88	21.6	70/142	49.3
	Low	27/220	12.3	16/167	9.6	11/52	21.1

BRD, bovine respiratory disease; cMICE, circular integrative and conjugative element.

Cattle at higher risk of developing clinical BRD during the feeding period had a higher cMICE percentage regardless of cattle type (Table 5). When compared to isolates from ranch direct-sourced calves, isolates from backgrounding dairy-type cattle carried more cMICE. However, the mean percentage of isolates with cMICE from beef cattle sourced from auction marts did not differ (*p =* 0.12) from beef cattle sourced from backgrounding operations.

**Table 5:** *Mycoplasmopsis bovis* cMICE in total samples, stratified by cattle type from various sources.

Risk factor	Risk factor levels	Percentage^*^	SE	95% CL	*p*-value^§^
Entire dataset					
Cattle type	Beef	9.8	±2.52	5.80, 15.94	<0.0001
	Dairy	44.5	±5.78	33.60, 55.96	
Beef					
Risk	High	15.2	±7.12	5.72, 34.76	0.0001
	Low	1.2	±0.77	0.35, 4.19	
Source	Auction	2.3	±1.46	0.65, 7.80	0.1202^¤^
	Backgrounding	9.7	±5.86	2.78, 28.66	
	Ranch direct	4.0	±2.17	1.36, 11.26	
Season	Fall	0.7	±0.73	0.07, 5.69	0.0020^¥^
	Spring	6.8	±2.65	2.92, 14.11	
	Winter	18.5	±5.96	9.47, 33.12	
Year	1st	1.7	±1.26	0.40, 7.05	0.0038
	2nd	11.3	±3.87	5.63, 21.42	
Dairy					
Risk	High	64.8	±10.50	42.60, 82.03	0.0241
	Low	31.0	±9.24	16.10, 51.33	
Origin	Backgrounding	70.6	±13.37	40.25, 89.52	0.0106
	Ranch direct	25.7	±5.19	16.79, 37.14	

^*^Percentage of cMICE-positivity. ^§^*p*-value of the comparison between the risk factor level group means of probability of cMICE-positivity. ^¤^*p*-value of the comparison between auction and backgrounding levels, it does not include ranch direct samples. ^¥^*p*-value of the comparison adjusted for multiple comparisons. CL, confidence limits; cMICE, circular integrative and conjugative element; SE, standard error of the mean.

### 3.6 Bacterial transformation

For the identification of *M. bovis* transconjugants, parental strains were tagged with ARGs (transformants). To eliminate the need for gDNA extraction prior to ARGs cPCR amplification, new direct-qPCR assays were developed. The new direct-qPCR assays designed for the detection of pMT85 antimicrobial resistance markers proved to be specific, with a dynamic range for pDNA and *M. bovis* transformants growing in broth that spanned several dilutions (Supplementary material 5). *M. bovis* 057 PURO- transformants, 643 GEN- transformants, and D317A TET- transformants were cPCR positive for the ARGs that were used to genetically mark them. Transformation was successful for 29/32 isolate-ARG combinations but failed for 13 isolate-ARG combinations (data not shown). For the isolates resulting in successful transformation, up to 9 transformants were stored per isolate-ARG combination. In summary, a total of 123 transformants were obtained belonging to 19 different isolate-antibiotic combinations (Supplementary material 3, Table S7).

### 3.7 Conjugation studies

From 20 independent conjugation experiments conducted using a previously published conjugation method referred to as the “*reference* conjugation method” (21), transconjugants were obtained in two instances (Supplementary material 8, Table S14). Those successful instances originated from conjugations between *M. bovis* isolate 643 (cMICE positive; contains two ICEB-2 and one ICEB-1 elements, Supplementary material 4, Figures S3, S4), and two independent recipient isolates *M. bovis* I100 (cMICE negative; its genome was not sequenced) and 057 (cMICE negative; contains one ICEB-1 element, Supplementary material 4, Figure S4). The rest of the suspected transconjugants obtained from the remaining 18 conjugation experiments were positive for just for one of the ARGs present in the parental isolates (i.e., PURO or GEN). These non-transconjugants were visible after 4–5 d and more frequently on SP4 + PURO+GEN agar plates incubated at 5% CO_2_ versus 0% CO_2_ (data not shown). Unlike non-transconjugants, most transconjugants were observed on SP4 + PURO+GEN plates only after 2 d of incubation and were PCR positive for both PURO and GEN.

Of the modifications to the r*eference* conjugation method, only orbital agitation of the bi-parent mixture consistently promoted conjugation (Supplementary material 8). Based on 3 different experiments with varying incubation time, the conjugation frequency between *M. bovis* 643^G^ and I100^P^ was optimal for incubations less than 10 h with agitation in SP4 broth (conjugation mean value ± standard deviation (x10^−6^): 11.2 ± 789.4) (Table 6).

**Table 6:** Conjugation frequencies between *M. bovis* 643^G^ and *M. bovis* I100^P^ with orbital shaking.

Experiment ID	O		P				S		
Inc. time (h)	5	10, 15, 20	2	4	6	8	1	2	4
Conjugation Frequency (%)	1.5 × 10^−7^	0	2.8 × 10^−7^	4.7 × 10^−8^	8.4 × 10^−8^	8.7 × 10^−8^	4.4 × 10^−8^	3.1 × 10^−8^	1.7 × 10^−7^

Experiment ID, see Supplementary Table S15 for conditions used in experiments O, P and S; h, hours; Inc., incubation.

## 4 Discussion

### 4.1 MICEs are widespread in Canadian *Mycoplasmopsis bovis*

Compared to ICEA_5632_-I, the H-type MICE identified in *M. bovis* exhibited structural differences that may have played a role in the enhanced conjugation efficiency observed with agitation. While CDSC has been described as part of the minimal ICEA_5632_ machinery, its function remains unknown (22). In our study, only approximately half of ICEA_5632_-I-CDSC length aligned with the CDS11 described in *M. bovis* MICE (data not shown). Likewise, ICEA_5632_-I-CDSH was found to be homologous to several bacterial DNA methyltransferases and hypothesized to be involved in the control of ICEA_5632_-I survival and propagation in various hosts by protecting transferred DNA from restriction enzymes (15). However, CDSH was absent in all of the *M. bovis* genomes screened. Two non-coding regions, *ncr16/27* and *ncrD/5*, previously described as possible regulatory and/ or *cis*-acting elements needed for conjugation (22) were also examined. The *ncr16/27* was found to be highly conserved in this study, which further supports its relevance in MICE. However, *M. bovis ncrD/5* differed in both size and sequence from that found in *M. agalactiae*. The H-Type CDS1 was found alone in 21.9% of the (draft and hybrid) assemblies in our study, and in all instances, it was associated with ISs, suggesting they may be remnants of an H-type MICE that degenerated over time.

Insertion sequences are important in bacterial evolution and are widespread in prokaryotes, but variation exists within and between species and frequently exhibit an evolutive history (36, 37). While IS have the ability to regulate transcription, their insertion within coding regions may interrupt gene function (38, 39). In our study, all H-type *M. bovis* MICE contained ISs either between or within CDSs (Supplementary material 4, Figure S3), whereas ICEA_5632_-I, II, and III did not (16). When *M. agalactiae* NCBI genomes (*n* = 10) were screened for IS in the ISFinder database (Aug 2024), with the exception of strain 5,632 (*n* = 28 complete IS), no more than 6 ISs were found per genome (data not shown). This contrasts with what has been described in *M. bovis* for which up to 103 ISs have been identified within a single genome (32). These results coincided with previous observations describing a higher IS density in *M. bovis* compared to *M. agalactiae* (40). Whether IS contribute to the reduced conjugation efficiency observed in *M. bovis* in SP4 broth as compared to *M. agalactiae* remains unclear due to the limited availability of *M. agalactiae* genomes with H-type MICE.

### 4.2 cMICE is positively associated to antimicrobial use in *Mycoplasmopsis bovis*

Although ICE circularization in bacteria does not guarantee conjugative transfer, PCR-detection of an ICE junction following chromosomal excision is frequently used as indirect evidence of ICE functionality (41). In our study, the overall percentage of cMICE-positive *M. bovis* isolates from the TMC collection (25.7%) was relatively close to the proportion of MJC isolates that carried all the MICE-CDSs required for conjugation in *M. agalactiae* 5,632 (33.1%), which could reflect the *in vivo* conjugation potential of *M. bovis*.

Dairy cattle were more positively associated to cMICE as compared to beef cattle. Additionally, within dairy, calves that originated from backgrounding operations were more likely to be cMICE-positive than those sourced directly from ranches. For beef cattle, backgrounded cattle tended to have more cMICE (9.7%) than auction-derived calves (2.3%), but this difference was not significant (*p* = 0.12). Given the low overall prevalence of cMICE in *M. bovis* from beef cattle (13.7%), power calculations indicated that a four-year sampling period would be required to detect statistically meaningful differences between sources at an 80% confidence level in beef cattle (data not shown). Antimicrobial use is generally higher in dairy farms than in the beef industry in western Canada (42, 43). Additionally, backgrounding operations, characterized by confinement and higher calf population density, are associated with increased antimicrobial use compared to ranches. Collectively, these findings suggest a correlation between elevated antimicrobial use and cMICE presence in *M. bovis* which could ultimately lead to the horizontal transmission of AMR in *M. bovis* populations. To date, the relationship between cMICE presence and AMR has not been directly investigated, but is currently being evaluated in our laboratory. Regardless of the cattle type, high risk calves were positively associated with cMICE as compared to low risk. The BRD risk level during the feeding period is determined by a series of animal and environmental factors such as age, weight, vaccination status, degree of commingling, transport distance, or extreme weather changes. The biological basis for the association between BRD risk status and the likelihood of isolating cMICE-positive *M. bovis* before the administration of antimicrobials at the feedlot remains unclear and warrants further investigation.

Interpretation of these epidemiological patterns should also consider broader contextual factors. Environmental and management-related variables, including antimicrobial use practices and co-infections, may influence cMICE distribution and warrant further exploration in future studies that integrate high-resolution genomic data with complementary management and environmental information. In addition, the binding sites of the newly developed qPCR primers (cMICE detection) were conserved across the hominis-type MICE identified in our isolates. However, the presence of additional MICE variants circulating within *M. bovis* populations that are not captured by the current direct-qPCR assay cannot be excluded.

### 4.3 *Mycoplasmopsis bovis* conjugates in SP4 broth

Two types of mycoplasma ICE have been described in previous research: hominis-type (H-type) and spiroplasma-type (S-type) (13). To-date, conjugation has only been demonstrated for H-type MICE, both in SP4 broth and cell culture (19), as the S-type MICE are typically degenerative and non-conjugative (23). In *M. agalactiae*, the horizontal transfer of MICE between donor and recipient cells has been documented, along with chromosomal transfer (CT) of random DNA fragments in the opposite direction (13). While CT uses MICE machinery, it occurs independently of MICE transfer (13). Importantly, CT can involve any region of the chromosome, including genes with mutations conferring enrofloxacin resistance, potentially contributing to the spread of AMR in mycoplasmas (20). More recently, H-type MICE transfer has also been documented between *M. bovis* strains in cell culture but failed in SP4 broth (19). In that study, *M. agalactiae* showed higher conjugation frequency in cell culture compared to axenic (SP4 broth) conditions. Moreover, *M. bovis* conjugation frequency in cell culture was similar to that in *M. agalactiae*, but the presence of CT between *M. bovis* cells was not determined.

Previous research has shown that shaking bacterial cultures can increase *E. coli* conjugation frequencies by up to 4 fold (44). In our study, shaking cultures in SP4 broth resulted in conjugation between *M. bovis* cells at comparable frequencies to those of *M. agalactiae* in SP4 broth (23). The presence of both parental antimicrobial markers (i.e., PURO and GEN) was confirmed by PCR using purified gDNA from transconjugants, suggesting the transfer of chromosomal fragments and/or MICE between cells as described in *M. agalactiae* (19). Therefore, a detailed genomic analysis of CT in *M. bovis* is warranted but falls outside the scope of this study. It is noteworthy that only those conjugation events that involved the transfer of the chromosomal region containing the ARG markers could be detected. Given that any genomic region has the potential to be horizontally transferred between mycoplasma cells (13), (undetected) chromosomal transfer events not involving the transfer of ARGs may have also taken place and have under estimated the conjugation frequency. Further investigation is needed to determine the degree that horizontal AMR transfer occurs in *M. bovis*.

The ruminants mycoplasma conjugation protocol does not specify the use of CO_2_ during the mating incubation. However, we selected a 5% CO_2_ atmosphere for conjugation experiments as this approximates its concentration in mammalian tissues (45), and is recommended for the isolation of mycoplasmas from ruminants (46). However, we observed a higher number of non-transconjugants *M. bovis* colonies (i.e., only PCR-positive for one of the ARG markers) on selective SP4 agar plates under 5% CO_2_. This may be due to CO_2_-induced acidification of media, which is known to impact the efficacy of penicillins, aminoglycosides, quinolones, macrolides, and tetracyclines (47). Therefore, we elected to not incubate SP4 agar containing antimicrobials under 5% CO₂ for selection of transconjugants, as a decrease in pH may reduce antimicrobial activity. Nevertheless, our high-throughput direct-qPCR assays designed for the detection of the antimicrobial markers may still be a valuable tool for screening suspected transconjugants grown with or without 5% CO_2_, as it eliminates the need for gDNA extraction.

The development of serum-free media for culturing mycoplasmas has been attempted in the past with limited success (48–50). More recently, laboratory-grown meat technologies have developed alternatives to FBS that support the growth of eukaryotic cells, like Proliferum (Multus Biotechnology. London, UK). When the suitability of Proliferum was tested in this study for the support of the *in vitro* growth of *M. bovis* in broth and agar, we obtained little success. However, other alternatives to FBS are now commercially available which warrant further investigation in the field. Ideally, a fully synthetic formula would provide greater consistency across mycoplasma media batches and eliminate the reliance on animal-derived components.

## 5 Conclusion

This study provides genomic and experimental evidence supporting the widespread presence and functionality of MICE in *M. bovis* isolates from Canadian feedlot cattle. Through whole-genome sequencing and cMICE screening, we confirmed the widespread presence of MICE-harboring *M. bovis* isolates, and importantly, demonstrated that these elements are capable of mediating conjugation *in vitro*. These findings emphasize the potential role of MICE in facilitating horizontal gene transfer, including the dissemination of AMR, and underscore their relevance in the epidemiology and evolution of *M. bovis*.

## Data Availability

The datasets presented in this study can be found in online repositories. All sequence read data from the current study were deposited in the NCBI database as Short Read Archive (SRA) under BioProject ID PRJNA1298945. Gene Bank https://www.ncbi.nlm.nih.gov/bioproject/PRJNA642970/ (BioProject ID PRJNA642970) (24).

## Glossary

AMR: antimicrobial resistance
ARG: antimicrobial resistance gene
BR: biological replicate
BRD: bovine respiratory disease
BUSCO: Benchmarking Universal Single-Copy *Orthologs*
CDS: Coding DNA Sequence
CFU: colony forming unit
cMICE: circular mycoplasma integrative and conjugative element
cPCR: conventional PCR
CT: chromosomal transfer
DNPS: deep nasopharyngeal swab sample
FBS: fetal bovine serum
gDNA: genomic DNA
GEN: gentamicin
HGT: horizontal gene transfer
H-MICE: hominis-type MICE
ICE: integrative and conjugative element
IME: integrative and mobilizable element
IR: inverted repeat
MGE: mobile genetic element
MICE: mycoplasma integrative and conjugative element
MJC: Murray Jelinski collection
pDNA: plasmid DNA
pMT85: plasmid pMT85
PURO: puromycin
qPCR: quantitative PCR
S-MICE: spiroplasma-type MICE
STC: same truck cluster
TET: tetracycline
TMC: Tim McAllister collection
Tn: transposon
vICE: vestigial ICE.
