# Correction: Integrative and conjugative elements in *Mycoplasmopsis bovis* from Western Canadian feedlot cattle: characterization and conjugative transfer

**DOI:** 10.3389/fvets.2026.1814107

**Published:** 2026-04-13

**Authors:** Sara Andres-Lasheras, Rahat Zaheer, Rodrigo Ortega-Polo, Timothy Schwinghamer, Sujeema Abeysekara, Athanasios Zovoilis, Sani-e-Zehra Zaidi, Murray Jelinski, Tim A. McAllister

**Affiliations:** 1 Lethbridge Research and Development Centre, Agriculture and Agri-Food Canada, Lethbridge, AB, Canada; 2 Department of Biochemistry and Medical Genetics, University of Manitoba, Winnipeg, MB, Canada; 3 Western College of Veterinary Medicine, University of Saskatchewan, Saskatoon, SK, Canada

**Keywords:** antimicrobial resistance, epidemiology, horizontal gene transfer, integrative and conjugative elements, *Mycoplasmopsis bovis*

The copyright for this article was incorrectly written as “© 2026 Andres-Lasheras, Zaheer, Ortega-Polo, Schwinghamer, Abeysekara, Zovoilis, Zaidi, Jelinski and McAllister. This is an open-access article distributed under the terms of the Creative Commons Attribution License (CC BY). The use, distribution or reproduction in other forums is permitted, provided the original author(s) and the copyright owner(s) are credited and that the original publication in this journal is cited, in accordance with accepted academic practice. No use, distribution or reproduction is permitted which does not comply with these terms.”

The correct statement is “Copyright © 2026 Athanasios Zovoilis, Sani e-Zehra Zaidi, Murray Jelinski, and His Majesty the King in Right of Canada, as represented by the Minister of Agriculture and Agri-Food Canada for the contribution of Sara Andres-Lasheras, Rahat Zaheer, Rodrigo Ortega Polo, Timothy Schwinghamer, Sujeema Abeysekara, and Tim McAllister. This is an open-access article distributed under the terms of the Creative Commons Attribution License (CC BY). The use, distribution or reproduction in other forums is permitted, provided the original author(s) and the copyright owner(s) are credited and that the original publication in this journal is cited, in accordance with accepted academic practice. No use, distribution or reproduction is permitted which does not comply with these terms.”

The original version of this article has been updated.

