# Supplementary material for: Integrative and conjugative elements in Mycoplasmopsis bovis from Western Canadian feedlot cattle: characterization and conjugative transfer
Source: Front Vet Sci. 2026 Jan 27;13:1719776. doi: 10.3389/fvets.2026.1719776 (PMC12892342; doi:10.3389/fvets.2026.1719776)
Supplement: Supplementary file 1 [file Data_Sheet_1.docx]

# 1. Mycoplasma isolates used in this study

**Supplementary Table S1.** Metadata of mycoplasma isolates used in this study

| **Isolate ID** | **Geo. origin** | **Year** | **Host** | **Feedlot** | **Cohort^1^ or truck load^2^** | **Animal number** | **Anatomical region** | **Health status** |
| --- | --- | --- | --- | --- | --- | --- | --- | --- |
| *Mycoplasmopsis bovis* 646 | Canada | 2007 | Feedlot cattle | F16 | C25 | 41 | DNPS | Healthy |
| *Mycoplasmopsis bovis* 630 | Canada | 2008 | Feedlot cattle | F8 | C15 | 44 | Joint | Dead |
| *Mycoplasmopsis bovis* C38 | Canada | 2017 | Feedlot cattle | C | C4 | 38 | DNPS | Healthy |
| *Mycoplasmopsis bovis* 643 | Canada | 2006 | Feedlot cattle | F20 | C29 | 9 | DNPS | Healthy |
| *Mycoplasmopsis bovis* J10 | Canada | 2017 | Feedlot cattle | J | J1 | 10 | DNPS | Healthy |
| *Mycoplasmopsis bovis* I44 | Canada | 2017 | Feedlot cattle | I | I1 | 44 | DNPS | Healthy |
| *Mycoplasmopsis bovis* I100 | Canada | 2017 | Feedlot cattle | I | NA | 100 | DNPS | Healthy |
| *Mycoplasmopsis bovis* G44 | Canada | 2017 | Feedlot cattle | G | G4 | 44 | DNPS | Healthy |
| *Mycoplasmopsis bovis* 057 | Canada | 2017 | Feedlot cattle | F12 | C19 | 118 | Lung | Dead |
| *Mycoplasmopsis bovis* J72 | Canada | 2018 | Feedlot cattle | J | J8 | 72 | DNPS | Healthy |
| *Mycoplasmopsis bovis* D317 | Canada | 2019 | Feedlot cattle | D | D20 | 317 | DNPS | Healthy |
| *Mycoplasmopsis bovis* C297 | Canada | 2019 | Feedlot cattle | C | C21.2 | 297 | DNPS | Healthy |
| *Mycoplasmopsis bovis* A120 | Canada | 2018 | Feedlot cattle | A | A12 | 120 | DNPS | Healthy |
| *Mycoplasmopsis bovis* J288 | Canada | 2019 | Feedlot cattle | J | J21 | 288 | DNPS | Healthy |
| *Mycoplasmopsis bovis* C176 | Canada | 2018 | Feedlot cattle | C | C13.3 | 176 | DNPS | Healthy |
| *Mycoplasmopsis bovis* 019 | Canada | 2016 | Feedlot cattle | F18 | C27 | 82 | Lung | Dead |
| *Mycoplasmopsis bovis* 064 | Canada | 2017 | Feedlot cattle | F1 | C3 | 96 | Joint | Dead |
| *Mycoplasmopsis bovis* 645 | Canada | 2007 | Feedlot cattle | F15 | C22 | 33 | DNPS | Diseased |
| *Mycoplasmopsis agalactiae* PG2 | Spain | 1952 | Caprine | NA | NA | NA | Unknown | Unknown |

^1^A cohort was a group of animals in the same feedlot at the same time (only applies to MJC collection). ^2^Transport trailer was considered the primary sampling unit, with cattle from the same truckload considered a cluster (only applies to TMC collection). DNPS, deep nasopharyngeal swab; Geo. Origin, geographical origin; NA; not applicable.

# 2. Mycoplasma culture

SP4 media has been used for mycoplasma conjugation experiments before (Supp. Table S2) (1). However, SP4 media can be expensive due to the supplements it contains such as fetal bovine serum (FBS). Consequently, different media were tested for their suitability in this and future studies i.e. supports conjugation, it is compatible with antimicrobial susceptibility testing (AST; i.e., it does not interact with the cell viability reagent alamarBlue used in our Laboratories (2)), and supports the growth of all the mycoplasma species of relevance in the feedlot cattle industry i.e. *M. bovis, M. dispar, M. bovirhinis, M. alkalescens, M. canadense, M. bovigenitalium, M. bovoculi, M. californicum, M. canis,* and *M. arginini* (3).

N broth was not suitable for ASTs since its base (brain heart infusion, BHI, (4)) reacted with alamarBlue (aB). This aB-BHI interaction generated a media color change characteristic of mycoplasma growth regardless there was bacteria growing in the broth (Supp. Table S3). Therefore, N media was discarded. PPLO did not react with aB (it has a heart infusion base instead of BHI) but did not support the growth of all species tested (data not shown). Additionally, when PPLO and Eaton’s were used for conjugation experiments following the reference method (1), no transconjugants were obtained (Supp. Table S13) which further lead to their dismissal. SP4 + FBS supported the growth of all species tested and did not interact with aB. Additionally, SP4 has been demonstrated to support sufficient *M. bovis* growth in broth microdilution ASTs harmonization studies (5).

**Supplementary Table S2.** SP4 media recipe

| **Component** | **Reference** | **For 1 L** |
| --- | --- | --- |
| *Mix together in a bottle and autoclave (121 °C, 15 min)* | | |
| PPLO broth | Difco, #255420 | 3.5 g |
| Bacto tryptone peptone | Gibco, #211705 | 10 g |
| Bacto peptone | Gibco, #211677 | 5.3 g |
| Select agar | Sigma, #A5054 | 9 g |
| Water | Invitrogen, #10977015 | 686 mL |
| pH | NaOH 5N | 7.8 |
| *Add aseptically* |  |  |
| Decomplemented FBS | Gibco, #12484028 | 170 mL |
| CMRL Medium (10X), no glutamine | Gibco, #21540026 | 50 mL |
| Bacto TC yeastolate, 4% w/v | Gibco, #255772 | 50 mL |
| Autolyzed yeast extract, 15% solution | Gibco, #18180059 | 25 mL |
| D (+) glucose (dextrose), 50% w/v | Sigma, #G8270 | 10 mL |
| Sodium pyruvate, 10% w/v | Sigma, #P5280 | 5 mL |
| Phenol red solution, 0.5% w/v* | Sigma, #P0290 | 4 mL |

FBS, fetal bovine serum. *Only for broth, not agar.

**Supplementary Table S3.** Different media tested in conjugation studies

| **Medium** | **aB compatible** | **Supports the growth of all mycoplasma spp. of interest** | **Supports**  ***M. bovis* conjugation** |
| --- | --- | --- | --- |
| N | No | ND | ND |
| PPLO | Yes | No | No* |
| Eaton’s | ND | ND | No* |
| SP4 + FBS | Yes | Yes | Yes |
| SP4 + Proliferum | ND | No | ND |

*Following the *reference* method without orbital agitation (see Supp. Material 6). aB, alamarBlue; PPLO, pleuropneumoniae-like microorganisms.

Additionally, Proliferum (PRF) was tested in SP4 broth as a synthetic alternative to FBS to determine its suitability for mycoplasma in vitro growth (Multus Biotechnology; London, UK). SP4 broth/ agar was prepared following standard procedures (1), split into 2 aliquots, and PRF or FBS were added to a final concentration of 17% v/v (1). *M. bovis* 646 field isolate stored at -80 °C in SP4+FBS at the logarithmic phase, was inoculated (1:100) into SP4-FBS and SP4-PRF and incubated overnight at 37 °C, 5% CO2 (Starter culture; 4 replicates). Each starter was subcultured (1:100) into the same type of broth (i.e. FBS into FBS, PRF into PRF) and monitored for up to 94.5 hr at different time intervals (range: 3 – 64.5 hr) in a Thermo Genesys 20 spectrophotometer, at 450 nm (6). Based on turbidity measurements, SP4+PRF did not support optimum *M. bovis* growth, therefore, further subcultures were carried out for verification purposes i.e. from SP4+FBS to SP4+FBS (optimum growth control; 2 replicates), SP4+PRF to SP4+FBS (growth restorage test; 1 replicates), and from SP4+FBS to SP4+PRF (growth performance test; 2 replicates). CFU enumerations on SP4+FBS agar were performed to further support broth culture turbidity observations. For this, 10-fold serial dilutions were carried out in DBPS + FBS 5% v/v (up to 10-7) and were spot-plated (10 µL of 10-5, 10-6, and 10-7 dilutions) onto agar. To test SP4+PRF agar suitability for *M. bovis* growth, the dilutions (from 10-1 to 10-7) corresponding to the “optimum growth control” subculture after 12 hr of incubation (mid-logarithmic phase), were plated onto SP4+FBS and SP4+PRF and incubated under the same conditions. To further determine whether 17 % v/v PRF was toxic to *M. bovis* 646 or not a sufficient concentration for optimum growth, a range of PRF concentrations were tested in SP4 broth i.e., 5%, 10%, 17%, 20%, 30%, and 50%. *Mycoplasmopsis bovis* 646 growth (37 °C, 5% CO2) was continuously and automatically monitored in a Stratus (Cerillo) every 30 min, at 450 nm, for 45 hr 30 min. SP4+FBS 17% v/v was used as a reference. Non-inoculated SP4 broth containing either FBS or PRF at 17% v/v were set as negative controls in this experiment.

Based on turbidity measurements from 4 independent replicates, SP4+PRF did not support optimum *M. bovis* growth (Supp. Table S4). Regardless of the inoculum origin, *M. bovis* 646 growth in SP4-FBS always showed a turbidity increase overtime (growth was restored in SP4-FBS when inoculated from SP4-PRF), whereas the growth in SP4-PRF barely presented an absorbance increase even when log-phase growth from SP4-FBS was used to inoculate it (Supp. Fig. S1). Absorbance results were further corroborated with CFU enumerations (Supp. Fig. S1).

**Supplementary Table S4.** Highest absorbance (450 nm) recorded across 4 independent replicates in SP4 broth

|  | **Subculture into** | |
| --- | --- | --- |
| **Inoculum origin (starter)** | **SP4-FBS** | **SP4-PRF** |
| SP4-FBS | 0.310 | NA |
| SP4-PRF | NA | 0.040 |

FBS, fetal bovine serum; NA, not applicable; PRF, Proliferum

**Supplementary Figure S1.** *Mycoplasmopsis bovis* 646 colony forming units per mL (CFU/mL) and absorbance in SP4 broth


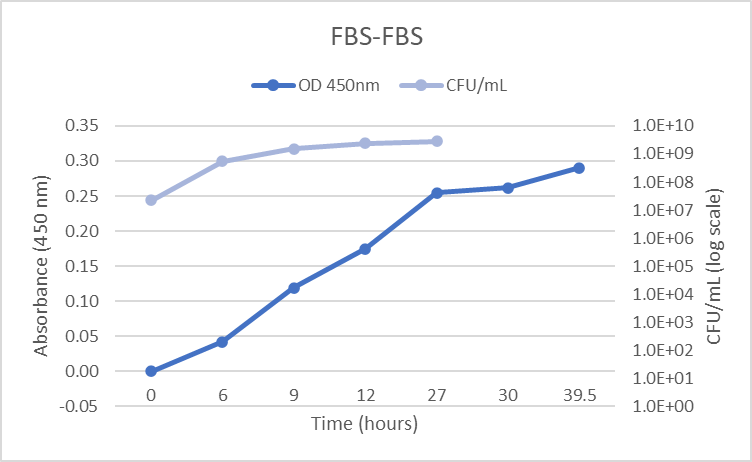


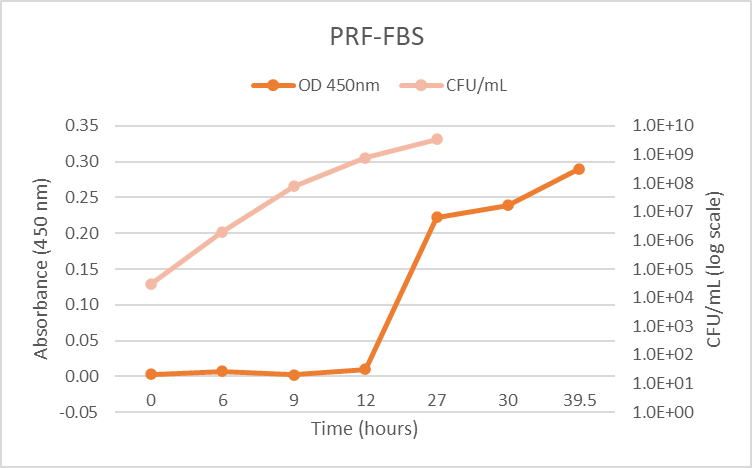


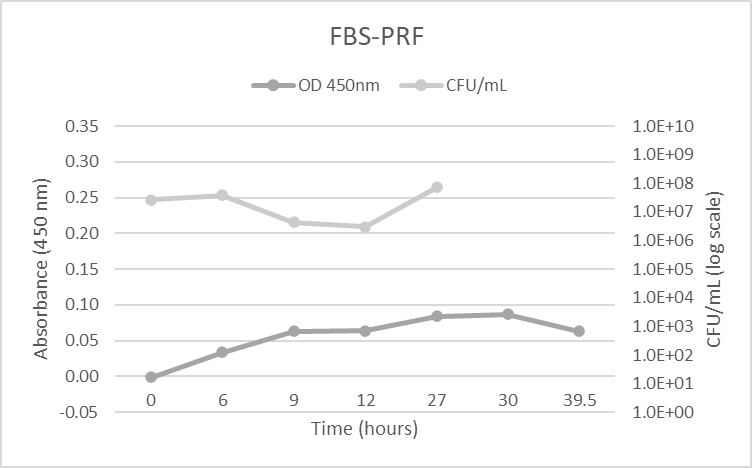


FBS-FBS, broth to broth subculture from SP4-FBS to SP4-FBS; PRF-FBS, broth to broth subculture from SP4- PRF to SP4- FBS, FBS-PRF, broth to broth subculture from SP4-FBS to SP4-PRF.

When log-phase, SP4+FBS growth was plated onto SP4+PRF agar (10-fold serial dilutions), individual colonies were only observed in dilution 10-1. However, the colony size was extremely small and presented an irregular size compared to *M. bovis* colonies in SP4 + FBS, therefore, they were not enumerated and SP4+PRF agar was deemed as not suitable for *M. bovis* growth. It should be noted that the 10-1 dilution of a mid-log phase growth of *M. bovis* 646 plated onto SP4 + FBS 17% v/v would have generated a bacterial lawn showing no individual countable colonies due to optimum growth performance. As shown in Supp. Fig. S2, none of the PRF concentrations tested in SP4 broth provided optimum in vitro growth of *M. bovis* 646 as compared to SP4 + FBS 17% v/v.

**Supplementary Figure S2.** Over time optical density (wavelength = 450 nm) of *Mycoplasmopsis bovis* 646 incubated in the portable turbidity reader Stratus (Cerillo) at 37 °C, humidity, and 5% CO_2_


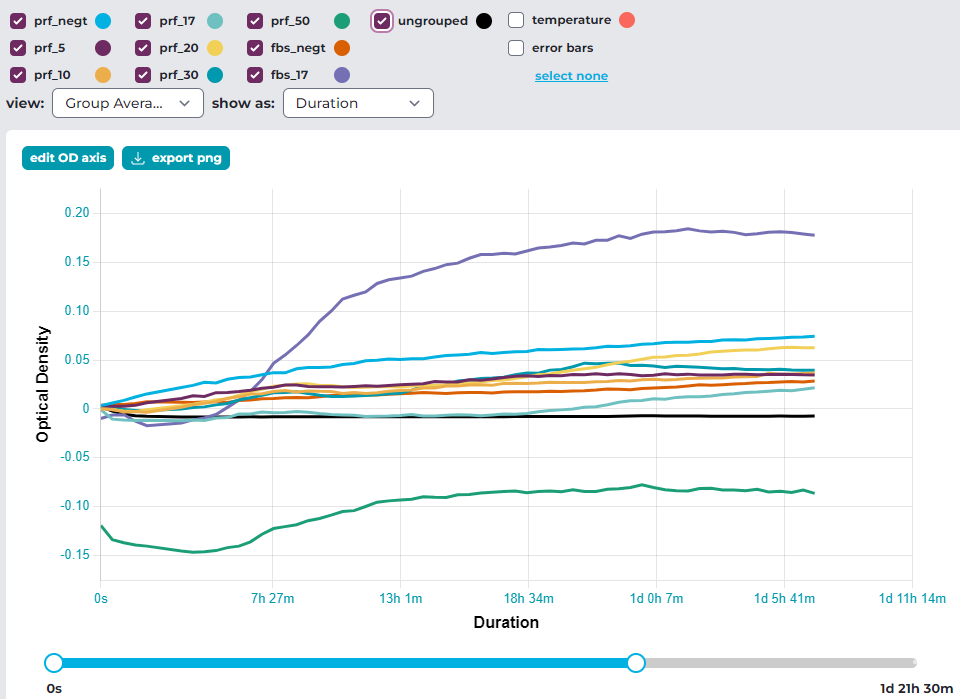


Screenshot from the Cerillo Labrador software (v2.3.3). fbs, fetal bovine serum; negt, negative; prf, Proliferum. Ungrouped: empty wells. Category numbers: PRF or FBS final concentration (% v/v) in SP4 broth. Optical density values correspond to the average of 3 technical replicates.

# 3. Genome sequencing and assembly quality parameters

**Supplementary Table S5.** *Mycoplasmopsis bovis* draft genomes used in this study

| **Isolates collection** | **No. of genomes sequenced** | **No. of genomes used in this study** | **No. of genomes re-sequenced^3^ (ID)** | **No. of genomes discarded (ID)** | **Total no. of genomes used in this study** |
| --- | --- | --- | --- | --- | --- |
| MJC | 126^1^ | 120 | 4^2^ (643, 645, 646, 019) | 2 (112, 060) | 124 |
| TMC | 3^2^ (J288, C176, J10) | 3 | NA | 0 | 3 |

**^1^**BioProject ID PRJNA642970; ^2^BioProject ID PRJNA1298945; ^3^Genomes were re-sequenced to increase coverage for the assembly of hybrid genomes. No, number.

**Supplementary Table S6.** *Mycoplasmopsis bovis* draft genome quality parameters generated by Quast (Galaxy Version 5.3.0+galaxy0)

| **Assembly** | **Average** | **Median** | **Range-Min** | **Range-Max** |
| --- | --- | --- | --- | --- |
| # contigs (>= 0 bp) | 212 | 180 | 123 | 488 |
| # contigs (>= 1000 bp) | 102 | 73 | 48 | 292 |
| Total length (>= 0 bp) | 952593 | 964107 | 686603 | 1041142 |
| Total length (>= 1000 bp) | 909756 | 926081 | 584356 | 996621 |
| # contigs | 127.72 | 89 | 64 | 427 |
| Largest contig | 58758.65 | 65109 | 9742 | 117120 |
| Total length | 928441.68 | 937898.5 | 673527 | 1006898 |
| GC (%) | 29.49 | 29.36 | 29.26 | 30.92 |
| N50 | 21391.26 | 23613.50 | 1952 | 45260 |
| N90 | 5315.60 | 5903.50 | 876 | 10491 |
| auN | 24247.73 | 27058 | 2634.20 | 43473.80 |
| L50 | 23.09 | 13.50 | 8 | 108 |
| L90 | 73.06 | 42 | 25 | 324 |
| # N's per 100 kbp | 0 | 0 | 0 | 0 |

**Supplementary Table S7.** Mycoplasma isolates used in this study, their characteristics, and number/ type of transformants

| **Isolate ID** | **Collection** | **cMICE** | **PURO-TF, colony #** | **GEN-TF, colony #** | **TET-TF, colony #** | **Short reads sequencing** | **Long reads sequencing** |
| --- | --- | --- | --- | --- | --- | --- | --- |
| *Mycoplasmopsis bovis* 646 | MJC | Post. | 5 | 8 | NA | Yes | Yes |
| *Mycoplasmopsis bovis* 630 | MJC | Post. | NA | 9 | 6 | Yes | Yes |
| *Mycoplasmopsis bovis* C38 | TMC | Post. | NA | 3 | NA | No | No |
| *Mycoplasmopsis bovis* 643 | TMC | Post. | 9 | 9 | NA | Yes | Yes |
| *Mycoplasmopsis bovis* J10 | TMC | Post. | NA | 9 | NA | Yes | Yes |
| *Mycoplasmopsis bovis* I44 | TMC | Post. | NA | 3 | NA | No | No |
| *Mycoplasmopsis bovis* I100 | TMC | Negt. | 9 | NA | NA | No | No |
| *Mycoplasmopsis bovis* G44 | TMC | Negt. | 9 | NA | NA | No | No |
| *Mycoplasmopsis bovis* 057 | MJC | Negt. | 9 | NA | NA | Yes | Yes |
| *Mycoplasmopsis bovis* J72 | TMC | Negt. | 9 | NA | NA | No | No |
| *Mycoplasmopsis bovis* D317 | TMC | Post. | NA | 8 | 9 | No | No |
| *Mycoplasmopsis bovis* C297 | TMC | Post. | 6 | 9 | NA | No | No |
| *Mycoplasmopsis bovis* A120 | TMC | Negt. | NA | NA | NA | No | No |
| *Mycoplasmopsis bovis* J288 | TMC | Negt. | NA | NA | NA | Yes | Yes |
| *Mycoplasmopsis bovis* C176 | TMC | Post. | NA | NA | NA | Yes | Yes |
| *Mycoplasmopsis bovis* 019 | MJC | Negt. | NA | NA | NA | Yes | Yes |
| *Mycoplasmopsis bovis* 064 | MJC | Negt. | NA | NA | NA | Yes | Yes |
| *Mycoplasmopsis bovis* 645 | MJC | Post. | NA | NA | NA | Yes | Yes |
| *Mycoplasmopsis agalactiae* PG2 | NA | ND | NA | 9 | NA | NA | NA |

cMICE, circular mycoplasma integrative and conjugative element; GEN, gentamicin; NA, not applicable; ND, not determined; MJC, Murray Jelinski *M. bovis* collection (2); Negt., negative; Post., positive; PURO, puromycin; TET, tetracycline; TF, transformant; TMC, Tim McAllister *M. bovis* collection (7).

**Supplementary Table S8.** *Mycoplasmopsis bovis* hybrid assembly quality parameters generated by Quast (*Mycovista*)

| **Assembly ID** | **# contigs (>= 0 bp)** | **# contigs (>= 1000 bp)** | **Total length (>= 0 bp)** | **Total length (>= 1000 bp)** | **# contigs** | **Largest contig** | **Total length** | **GC (%)** | **N50** | **N90** | **auN** | **L50** | **L90** | **# N's per 100 kbp** |
| --- | --- | --- | --- | --- | --- | --- | --- | --- | --- | --- | --- | --- | --- | --- |
| 646 | 1 | 1 | 1057333 | 1057333 | 1 | 1057333 | 1057333 | 29.23 | 1057333 | 1057333 | 1057333 | 1 | 1 | 0 |
| 645 | 1 | 1 | 1084262 | 1084262 | 1 | 1084262 | 1084262 | 29.21 | 1084262 | 1084262 | 1084262 | 1 | 1 | 0 |
| 643 | 1 | 1 | 1090990 | 1090990 | 1 | 1090990 | 1090990 | 29.16 | 1090990 | 1090990 | 1090990 | 1 | 1 | 0 |
| 630 | 1 | 1 | 1154934 | 1154934 | 1 | 1154934 | 1154934 | 29.08 | 1154934 | 1154934 | 1154934 | 1 | 1 | 0 |
| 064 | 1 | 1 | 1017720 | 1017720 | 1 | 1017720 | 1017720 | 29.27 | 1017720 | 1017720 | 1017720 | 1 | 1 | 0 |
| 057 | 3 | 3 | 1068896 | 1068896 | 3 | 1050361 | 1068896 | 29.21 | 1050361 | 1050361 | 1032346 | 1 | 1 | 0 |
| 019 | 3 | 3 | 1071125 | 1071125 | 3 | 1066033 | 1071125 | 29.39 | 1066033 | 1066033 | 1060981 | 1 | 1 | 0 |
| J288 | 1 | 1 | 1008447 | 1008447 | 1 | 1008447 | 1008447 | 29.25 | 1008447 | 1008447 | 1008447 | 1 | 1 | 0 |
| J10 | 4 | 4 | 1044142 | 1044142 | 4 | 976369 | 1044142 | 29.26 | 976369 | 976369 | 916913 | 1 | 1 | 0 |
| C176 | 1 | 1 | 1069210 | 1069210 | 1 | 1069210 | 1069210 | 29.27 | 1069210 | 1069210 | 1069210 | 1 | 1 | 0 |

**Supplementary Table S9.** *Mycoplasmopsis bovis* hybrid assembly quality parameters generated by BUSCO (v5.8.0)

| **Assembly ID** | **C176** | **J10** | **J288** | **019** | **057** | **064** | **630** | **643** | **645** | **646** |
| --- | --- | --- | --- | --- | --- | --- | --- | --- | --- | --- |
| Complete BUSCOs | 173 | 171 | 171 | 173 | 172 | 170 | 172 | 171 | 170 | 168 |
| Complete and single-copy BUSCOs | 172 | 171 | 170 | 173 | 172 | 170 | 172 | 171 | 170 | 168 |
| Complete and duplicated BUSCOs | 1 | 0 | 1 | 0 | 0 | 0 | 0 | 0 | 0 | 0 |
| Fragmented BUSCOs | 1 | 1 | 3 | 1 | 2 | 2 | 2 | 3 | 4 | 6 |
| Missing BUSCOs | 0 | 2 | 0 | 0 | 0 | 2 | 0 | 0 | 0 | 0 |
| Total BUSCO groups searched | 174 | 174 | 174 | 174 | 174 | 174 | 174 | 174 | 174 | 174 |

# 4. Comparative analysis of the MICE structure

**Supplementary Table S10**. Mycoplasma isolates used in this study for a comparative analysis of the MICE structure

| **Isolate ID** | **Reference** |
| --- | --- |
| 019, 630, 643, 645, 646, 057, 067, C176, J10, J288 | This study |
| F9160, L15527, L15762 | Ambroset et al. (2022) (8) |
| J279, J228, J137, J81, J6, RM16 | Garcia-Galan et al. (2022) (9) |
| 13DD0918, 14DD0147, 14DD0475, 15DD0123, 15DD0141, 15DD0160, 15DD0161, 15DD0165, 15DD0207, 15DD0210, 15DD0228, 15DD0233, 15DD0234, 15DD0238, 15DD0240, 15DD0249, 15DD0250, 15DD0261, 15DD0124, 16DD0001, 16DD0054, 16DD0100 | Triebel et al. (2023) (10) |

**Supplementary Figure S3**. Hominis-type mycoplasma MICE structure


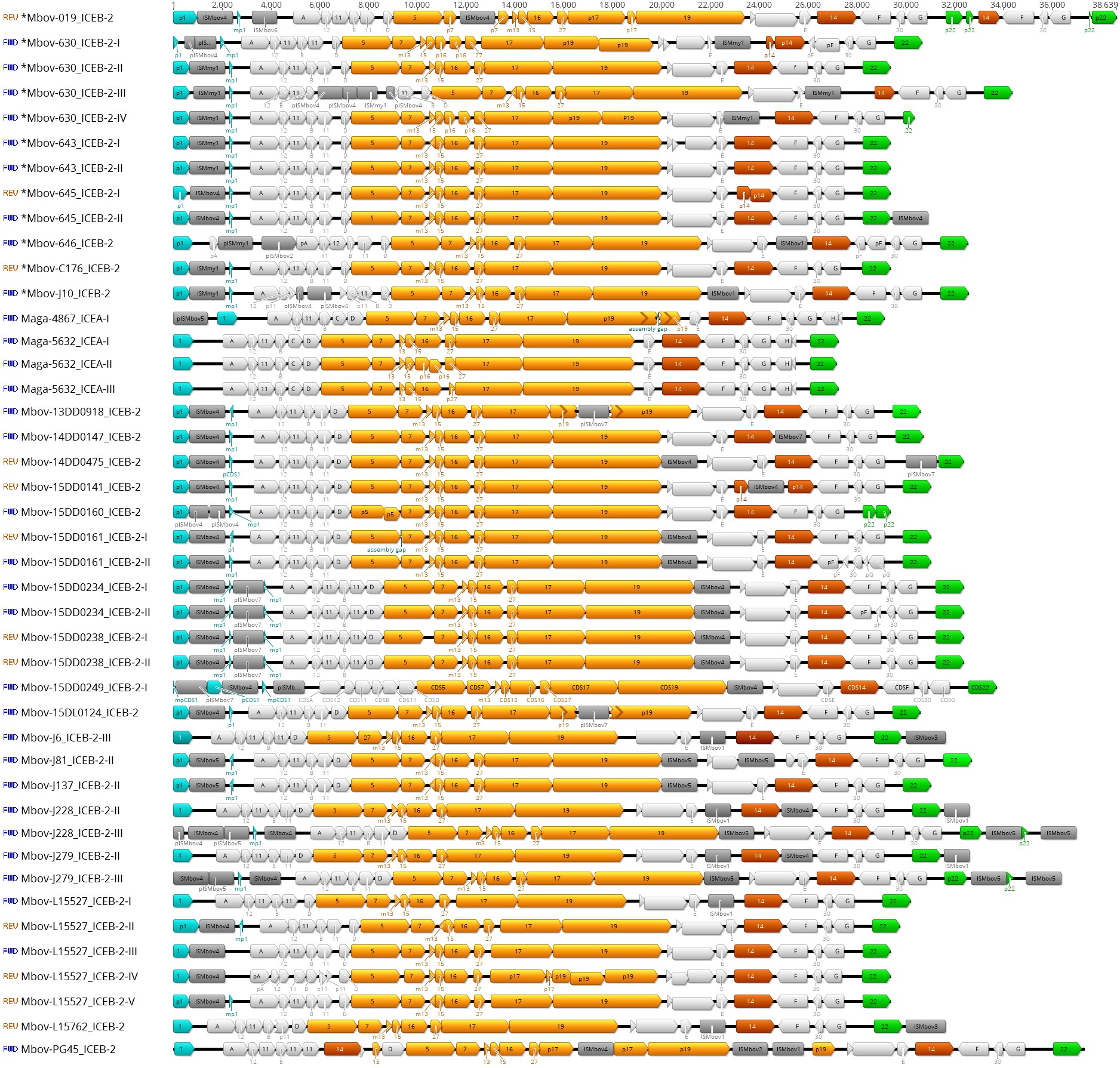


**Supplementary Figure S3**. Hominis-type mycoplasma MICE structure (continue)


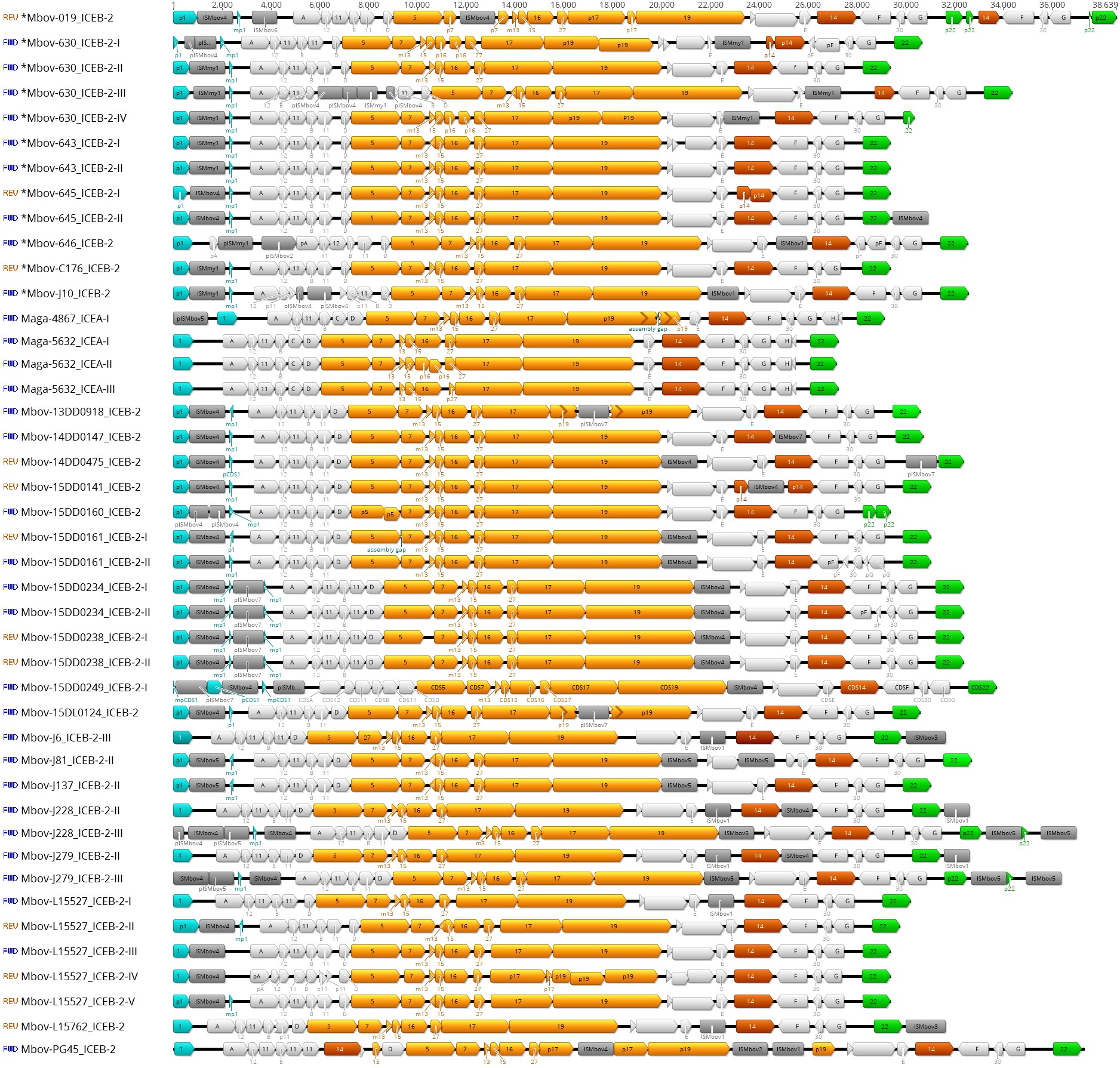


Green arrows, transposase; light orange, CDS candidates for conjugative channel (CDS5-19); dark orange, CDS14 that has a key role in ICE and chromosome transfer (11); dark grey, insertion sequences (IS). Top numbers, size in bp; *field isolates from this study; m, manually added/annotated CDS; p, partial gene (split or incomplete) or pseudogene. MICE sequence sources: Ambroset et al. (2022), Garcia-Galan et al. (2022), and Triebel et al. (2023); *Mycoplasmopsis agalactiae* (Maga) 5632 and 4867 (CT030003.1 and GCA_009150585.1, respectively), and *Mycoplasmopsis bovis* (Mbov) PG45 (NC_014760.1).

**Supplementary Figure S4**. Spiroplasma-type mycoplasma MICE structure


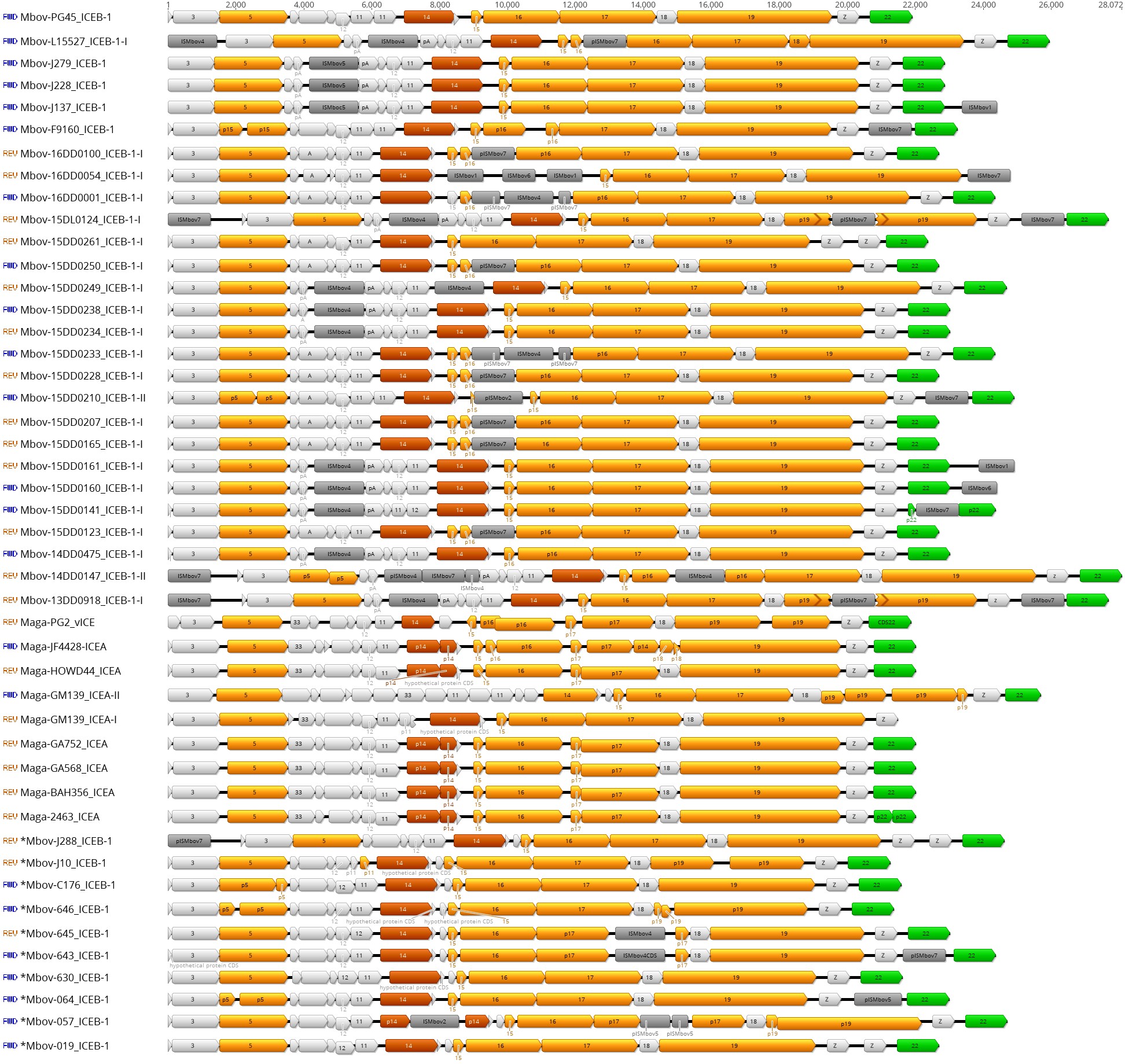


**Supplementary Figure S4**. Spiroplasma-type mycoplasma MICE structure (continue)


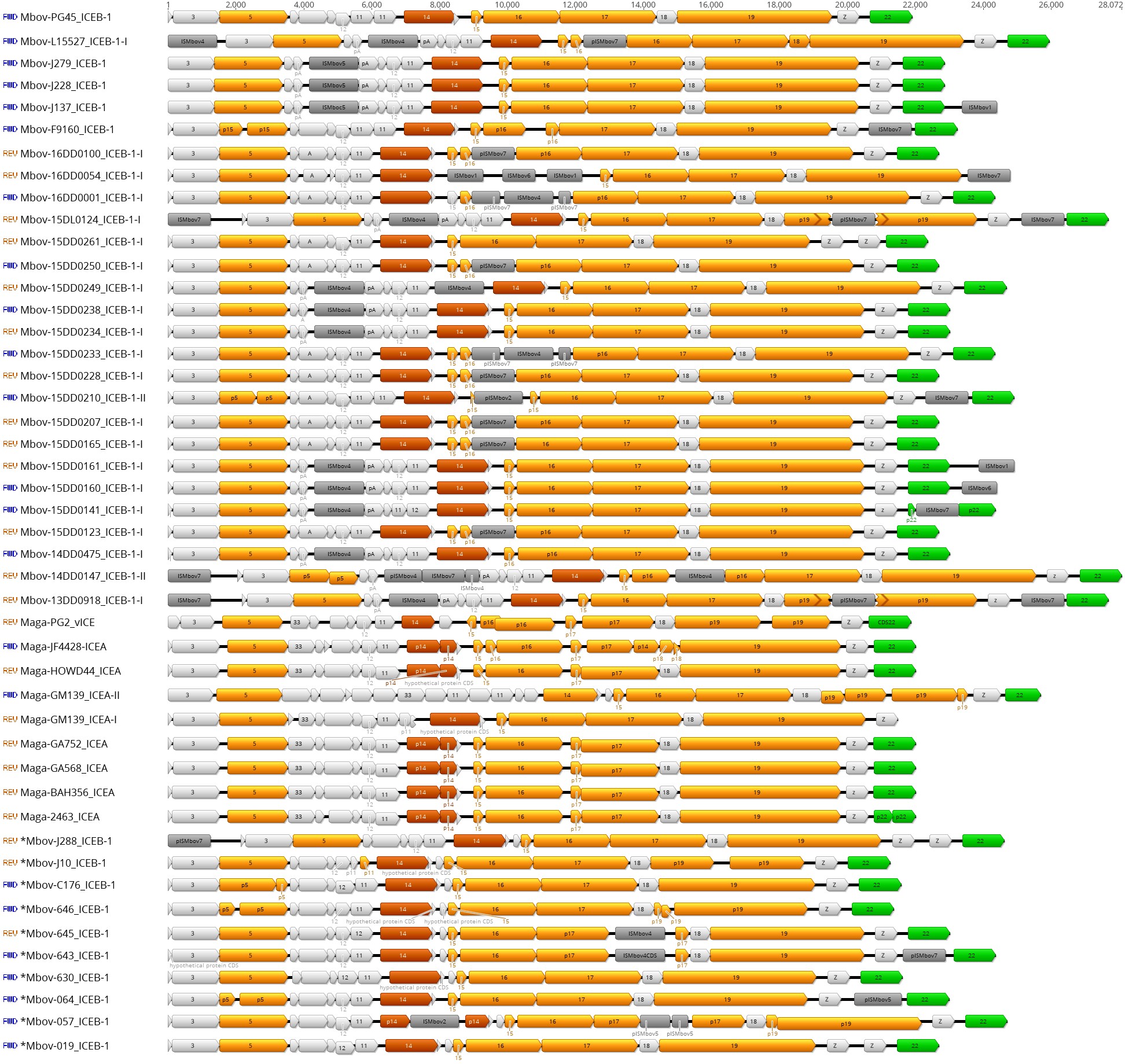


Green arrows, transposase; light orange, CDS candidates for conjugative channel (CDS5-19); dark orange, CDS14 that has a key role in ICE and chromosome transfer (11); dark grey, insertion sequences (IS). Top numbers, size in bp; *field isolates from this study; m, manually annotated CDS; p, pseudogene or gene split into two different CDSs. MICE sequence sources: Ambroset et al. (2022), Garcia-Galan et al. (2022), and Triebel et al. (2023); *Mycoplasmopsis agalactiae* PG2 (GCA_000063605.1) and *Mycoplasmopsis bovis* PG45 (NC_014760.1).

# 5. Quantitative PCR development and characteristics

***Detection of antimicrobial resistance markers for puromycin, gentamicin, and tetracycline***

The specificity of the new antimicrobial resistance marker primers was verified as follows: each pair of primers were tested on I) vector DNA (vDNA) corresponding to all the pMT85 derivative plasmids containing the ARGs (cPCR using vDNA at 0.5 ng/µL); and II) non-TF, non-TC *M. bovis* growing in SP4 (direct-qPCR). Likewise, the sensitivity of the new ARG qPCR assays was determined: 10-fold serial dilutions of I) vDNA by qPCR (from 5 to 0.5 x10-7 ng/µL); and II) transformed *M. bovis* growing in SP4 broth by direct-qPCR (109 – 102 CFU/mL) (Supp. Table S10). For the direct-qPCR approach, 2 µL of a 24 h-old *M. bovis* broth culture (109 CFU/mL) were directly used as the DNA template by increasing from 3 to 8 min the initial denaturation time (Supp. Table S12). Each experiment was repeated 3 independent times, and each dilution was tested in triplicates by qPCR. For qPCR assays, the SsoAdvanced Universal SYBR Green Supermix from Bio-Rad and an ABI StepOnePlus qPCR thermocycler were used. To carry out cPCR experiments, the HotStarTaq Plus Master Mix Kit (Qiagen) and an Eppendorf Flexlid Mastercycler nexus eco were used.

***Detection of cMICE***

New qPCR primers were designed in Geneious (v10.2.6) and their suitability to amplify the cMICE was determined by cPCR and Sanger sequencing (Eurofins Genomics). Once the new primers specificity was confirmed, the new qPCR dynamic range and LOD were determined for direct-qPCR (*M. bovis* 646 cultured in SP4 broth) and standard qPCR using purified gDNA (DNeasy Blood & Tissue DNA isolation kit; Qiagen) (12). For the direct-qPCR, 2 µL of *M. bovis* 646 growing in SP4 broth were used as the DNA template as follows: after 24 h of incubation at 37 °C and 5% CO2, *M. bovis* 646 growth (109 CFU/mL) was 10-fold serially diluted (up to 6 dilutions), enumerated on agar, and tested for cMICE by qPCR. The experiment was repeated 3 independent times, and each dilution was tested in triplicates by qPCR. To carry out cPCR experiments, the HotStarTaq Plus Master Mix Kit (Qiagen) and an Eppendorf Flexlid Mastercycler nexus eco were used.

**Supplementary Table S11.** Quantitative PCR characteristics for the detection of the plasmid pMT85 derivatives and cMICE

| **Target** | **Assay dynamic range and LOD** | **Efficiency** | **Slope** | **R^2^ value** | **Efficiency E*** |
| --- | --- | --- | --- | --- | --- |
| PURO | vDNA: 10^-01^ – 10^-06^ | 100.672 | -3.307 | 0.999 | 2.007 |
|  | Bacteria: 10^+09^ – 10^+04^ | 114.847 | -3.019 | 0.998 | 2.148 |
| GEN | vDNA: 10^-01^ – 10^-06^ | 97.245 | -3.390 | 0.999 | 1.972 |
|  | Bacteria: 10^+09^ – 10^+03^ | 97.897 | -3.373 | 0.999 | 1.979 |
| TET | vDNA: 10^-01^ – 10^-06^ | 97.013 | -3.396 | 1.000 | 1.970 |
|  | Bacteria: 10^+09^ – 10^+04^ | 118.192 | -2.965 | 0.955 | 2.182 |
| cMICE | gDNA: 10^+01^ – 10^-02^ | 95.038 | -3.449 | 0.999 | 1.950 |
|  | Bacteria: 10^+09^ – 10^+07^ | 112.528 | -3.105 | 0.983 | 2.125 |

Dynamic range expressed in ng/µL for vDNA and gDNA, and in CFU/mL for *M. bovis* liquid culture (Bacteria). cMICE, circular mycoplasma integrative and conjugative element; LOD, limit of detection; MC, melt curve; vDNA, vector DNA. qPCR parameters are averaged values from 3 different experiments. *Efficiency value (E) as per Pfaffl (2001).

# 6. Circular MICE, environmental factors

cMICE was quantified in *M. bovis* 646 under the following conditions: growth phase at 37 °C and 5% CO2 (early log phase (6 h) as a reference compared to late log (15 h), and early (20 h) and late (25 h) stat phase), cold (1h exposure to cold; 37 °C as a reference vs 4 °C), higher cell density (1h exposure to higher density; non-concentrated growth as a reference vs 2 mL growth concentrated into 900 uL of SP4), heat (1h exposure; 37 °C as a reference vs 45 °C), pH (1h exposure; 7.8 as a reference vs 4 and 9), starvation (1h exposure; SP4 broth as a reference vs DPBS), UV light (non-exposure as a reference vs 100 J/m^2^) (13), exposure to sub-inhibitory concentrations of mitomycin C (MMC; no MMC as a reference vs 0.016 and 0.032 μg/mL; 2.5h exposure), a combination of starvation + cold + higher cell density (1h exposure) followed by 1h (S4D-1h) or overnight (S4D-OV) (14) incubation under standard growing conditions (37 °C, 5% CO2, static), and atmosphere (5% CO2 as a reference vs 0%, at the logarithmic and early stationary phase). Samples were processed immediately after treatment.

**Supplementary Table S12.** *Mycoplasmopsis bovis* 646 enumerations before and after exposure to different environmental conditions for the quantification of cMICE

|  | Control sample before treatment (CFU/mL) | Control sample after treatment (CFU/mL) | Treatment sample after treatment (CFU/mL) |
| --- | --- | --- | --- |
| UV light, 100 J/m^2^ | 5.50E+08 | 9.23E+08 | 2.97E+08 |
| Higher cell density | 1.43E+09 | 1.50E+09 | 1.40E+09 |
| Cold, 4 °C | 2.31E+09 | 3.56E+09 | 2.27E+09 |
| Starvation, DPBS | 1.90E+09 | 2.43E+09 | 2.17E+09 |
| Heat, 45 °C | 1.34E+09 | 1.54E+09 | 1.48E+09 |
| SD4 – 1hr | 2.16E+09 | 2.63E+09 | 1.72E+09 |
| SD4 – OV | 2.16E+09 | 7.79E+09 | 6.27E+09 |
| pH = 5 | 2.39E+09 | 3.30E+09 | 9.40E+08 |
| pH = 9 | 2.39E+09 | 3.30E+09 | 1.73E+09 |
| MMC, 0.016 µg/mL | 1.77E+09 | 4.94E+06* | 3.59E+06 |
| MMC, 0.032 µg/mL | 1.77E+09 | 4.94E+06* | 4.81E+06 |
| Growth phase, early log | 1.37E+09 | NA | NA |
| Growth phase, late log | 3.51E+09 | NA | NA |
| Growth phase, early stat | 3.96E+09 | NA | NA |
| Growth phase, late stat | 3.62E+09 | NA | NA |
| Atmosphere, CO_2_ log | 2.87E+09 | NA | NA |
| Atmosphere, CO_2_ stat | 4.19E+08 | NA | NA |
| Atmosphere, O_2_ log | 2.80E+09 | NA | NA |
| Atmosphere, O_2_ stat | 4.30E+08 | NA | NA |

CFU, colony forming units; DPBS, Dulbecco’s phosphate buffered saline; log, logarithmic; MMC, mitomycin C; OV, overnight; SD4, combination of starvation + higher cell density + 4 °C; stat, stationary. *Bacterial inoculum were diluted to 1E+06 to test MMC according to standard susceptibility testing procedures (2).

# 7. Primers used in this study and PCR cycling conditions

**Supplementary Table S13.** Primers used in this study

| **Name** | **Target** | **Sequence (5’ – 3’)** | **PCR type** | **Amplicon size (bp)** | **Cycling conditions** | **Reference** |
| --- | --- | --- | --- | --- | --- | --- |
| PURO-F | PURO | GTTGCTGATGATGGTGCTGC | qPCR | 74 | 98 °C, 3^¤^ min;  [98 °C, 30 sec; 60 °C, 30 sec] x 35 cycles  MC: 65 – 95 °C; fluorescence measured every 0.2 °C; step and hold | This study |
| PURO-R |  | TCAGCAAAAACAGCACCAGC |  |  |  |  |
| GEN-F | GEN | CCAAGAGCAATAAGGGCATACC | qPCR | 76 |  | This study |
| GEN-R |  | CCTCGTGTAATTCATGTTCTGGC |  |  |  |  |
| TET-F | TET | TCCCTCTTTATCATGGAAGTGCA | qPCR | 100 |  | This study |
| TET-R |  | GACGGACCTCGATGTGTTGA |  |  |  |  |
| purF | PURO | GTTGCTGTTTGGACTACTCCTG | cPCR | 359 | 95 °C, 5 min;  [94 °C, 30 sec, 52 °C (PURO), 55 °C (GEN), 57.5 °C (TET), 30 sec, 72 °C, 30 sec] x 35 cycles  72 °C, 10 min | (15) |
| purR |  | CACCAAGTTCTAGGACCTTCAGG |  |  |  |  |
| Gm1 | GEN | ACATGAATTACACGAGGGC | cPCR | 410 |  | (15) |
| Gm2 |  | GTTCTTCTTCTGACATAGTAG |  |  |  |  |
| IntMtet1 | TET | TGGCGTACAAGCACAAACTC | cPCR | 444 |  | (15) |
| IntMtet2 |  | GCAAAGTTCAGACGGACCTC |  |  |  |  |
| Left1 | cMICE | TAATGGCCAAGAGTTCAAAAGCAA | cPCR | ~650 | 95 °C, 5 min;[94 °C, 30 sec; 58 °C, 30 sec; 72 °C, 30 sec] x 35 cycles; 72 °C, 10 min | (15) |
| Right2 |  | TACACAAGTGGTAATGCTGAAACA |  |  |  |  |
| cMICE-F | cMICE | TCTTATGCATAGAAGTAAAGTAGAGT | qPCR | 115 | 98 °C, 3^¤^ min; [98 °C, 30 sec; 60 °C, 30 sec] x 35 cycles. MC: 65 – 95 °C; fluorescence measured every 0.2 °C; step and hold | This study |
| cMICE-R |  | ACCCACTTTCTTCTATCAGTTC |  |  |  |  |
| uvrC-F | *uvrC* | CCTGTCGGAGTTGCAATTGT | qPCR | 92 | 98 °C, 3^¤^ min; [98 °C, 30 sec; 60 °C, 30 sec] x 35 cycles. MC: 65 – 95 °C; fluorescence measured every 0.3 °C; step and hold | (12) |
| uvrC-R |  | GCACTGCGCTCATTTAAAGC |  |  |  |  |

CDS, coding sequence; cMICE, circular mycoplasma integrative and conjugative element; cPCR, conventional PCR; GEN, gentamicin; MC, melt curve; PURO, puromycin; qPCR, quantitative PCR; TET, tetracycline. ^¤^increase to 8 min when using bacterial liquid growth as the DNA template as opposed to purified gDNA.

# 8. Conjugation experiments

**Supplementary Table S14.** Mycoplasma troubleshooting conjugation experiments carried out in this study

| **Round** | **Parent A** | **Parent B** | **Method** | **CO_2_ %** | **Isolates ratio A:B** | **Inc. time (hr)** | **Mating vol^¤^ (mL)** | **Plated vol* (mL)** | **Media** | **TC** |
| --- | --- | --- | --- | --- | --- | --- | --- | --- | --- | --- |
| 1 | Mb p646^P^ (3) | Maga pPG2^G^ (19) | Reference | 5 | 1:1 | 16, 20, 24 | 1 | 0.9 | SP4 | Absent |
| 2 | Mb p646^P^ (3) | Maga pPG2^G^ (19) | Reference | 5 | 1:10 | 16, 20, 24 | 1 | 0.9 | SP4 | Absent |
| A | Mb p057^P^ (9) | Mb p630^G^ (9) | Reference | 5 | 1:1 | 24 | 1 | 0.9 | SP4 | Absent |
| A | Mb p057^P^ (9) | Mb pC38 ^G^ (3) | Reference | 5 | 1:1 | 24 | 1 | 0.9 | SP4 | Absent |
| A | Mb p057^P^ (9) | Mb p643^G^ (9) | Reference | 5 | 1:1 | 24 | 1 | 0.9 | SP4 | Present |
| A | Mb p057^P^ (9) | Mb p646^G^ (9) | Reference | 5 | 1:1 | 24 | 1 | 0.9 | SP4 | Absent |
| A | Mb p057^P^ (9) | Mb pI44^G^ (3) | Reference | 5 | 1:1 | 24 | 1 | 0.9 | SP4 | Absent |
| A | Mb pI100^P^ (9) | Mb p630^G^ (9) | Reference | 5 | 1:1 | 24 | 1 | 0.9 | SP4 | Absent |
| A | Mb pI100^P^ (9) | Mb pC38 ^G^ (3) | Reference | 5 | 1:1 | 24 | 1 | 0.9 | SP4 | Absent |
| A | Mb pI100^P^ (9) | Mb p643^G^ (9) | Reference | 5 | 1:1 | 24 | 1 | 0.9 | SP4 | Present |
| A | Mb pI100^P^ (9) | Mb p646^G^ (9) | Reference | 5 | 1:1 | 24 | 1 | 0.9 | SP4 | Absent |
| A | Mb pI100^P^ (9) | Mb pI44^G^ (3) | Reference | 5 | 1:1 | 24 | 1 | 0.9 | SP4 | Absent |
| B | Mb pG44^P^ (9) | Mb p630^G^ (9) | Reference | 5 | 1:1 | 24 | 1 | 0.9 | SP4 | Absent |
| B | Mb pG44^P^ (9) | Mb pC38 ^G^ (3) | Reference | 5 | 1:1 | 24 | 1 | 0.9 | SP4 | Absent |
| B | Mb pG44^P^ (9) | Mb p643^G^ (9) | Reference | 5 | 1:1 | 24 | 1 | 0.9 | SP4 | Absent |
| B | Mb pG44^P^ (9) | Mb p646^G^ (9) | Reference | 5 | 1:1 | 24 | 1 | 0.9 | SP4 | Absent |
| B | Mb pG44^P^ (9) | Mb pI44^G^ (3) | Reference | 5 | 1:1 | 24 | 1 | 0.9 | SP4 | Absent |
| B | Mb pJ72^P^ (9) | Mb p630^G^ (9) | Reference | 5 | 1:1 | 24 | 1 | 0.9 | SP4 | Absent |
| B | Mb pJ72^P^ (9) | Mb p643^G^ (9) | Reference | 5 | 1:1 | 24 | 1 | 0.9 | SP4 | Absent |
| B | Mb pJ72^P^ (9) | Mb p646^G^ (9) | Reference | 5 | 1:1 | 24 | 1 | 0.9 | SP4 | Absent |
| C | Mb p057^P^ (9) | Mb p643^G^ (9) | Reference modified | 5 | 1:1 | 8, 16, 24 | 0.1, 0.5 | 0.9 | SP4 | Absent |
| D | Mb p057^P^ (9) | Mb p643^G^ (9) | Reference modified | 0, 5 | 1:1 | 24 | 1 | 0.9 | SP4 | Absent |
| E | Mb p057^P^ (9) | Mb i643^G^ (9) | Reference modified | 0 | 1:1 | 24 | 1 | 0.9 | SP4 | Absent |
| E | Mb pI100^P^ (9) | Mb i643^G^ (9) | Reference modified | 0 | 1:1 | 24 | 1 | 0.9 | SP4 | Absent |
| F | Mb p057^P^ (9) | Mb p643^G^ (9) | Reference modified | 0 | 1:10  1:100 | 24 | 1 | 0.9 | SP4 | Absent |
| F | Mb pI100^P^ (9) | Mb p643^G^ (9) | Reference modified | 0 | 1:10  1:100 | 24 | 1 | 0.9 | SP4 | Absent |
| G | Mb p057 ^P^ (9) | Mb p643^G^ (9) | Reference | 0 | 1:1 | 24 | 1, +Amp | 0.9 | SP4 | Absent |
| G | Mb pI100^P^ (9) | Mb p643^G^ (9) | Reference | 0 | 1:1 | 24 | 1, +Amp | 0.9 | SP4 | Absent |
| H | Mb pI100^P^ (9) | Mb p643^G^ (9) | Reference | 0 | 1:1 | 24 | 4 | 4 | SP4 | Absent |
| I | Mb p643^P^ (9) | Mb p643^G^ (9) | Reference | 0 | 1:1 | 24, 48 | 1 | 0.9 | SP4 | Absent |
| I | Mb pC297^P^ (6) | Mb pC297^G^ (9) | Reference | 0 | 1:1 | 24, 48 | 1 | 0.9 | SP4 | Absent |
| J | Mb pI100^P^ (9) | Mb p643^G^ (9) | Reference | 0 | 1:1 | 3, 6, 9, 12, 15, 18 | 1 | 0.9 | SP4 | Absent |
| K | Mb pI100^P^ (9) | Mb p643^G^ (9) | Reference modified | 0 | 1:1 | 24 | 1 | 0.9 | Eaton’s  PPLO | Absent |
| L | Mb p646^P^ (5) | Mb p646^G^ (8) | Reference modified | 0 | 1:1 | 24 | 1 | 0.9 | SP4 | Absent |

^¤^Fresh liquid media volume used to resuspend the bacterial pellet originated after centrifugation of 2 mL of parent A+B mixed culture.

*Conjugation broth volume plated per incubation time onto SP4 agar supplemented with antimicrobials for the detection of transconjugants. Antibiotic resistance markers harboured by the transformed parent strain are indicated as superscripted p (PURO) and g (GEN).

(number in brackets), Number of transformants used in each mating experiment; i, individual transformant clones; p, pool of transformants as prefix.

In the following experiments, a modified *reference*- protocol was followed, a parents’ ratio was kept at 1:1, and only SP4 media was used:

**Supplementary Table S15.** Mycoplasma conjugation experiments carried out in this study and their characteristics

| **Experiment** | **Parent A** | **Parent B** | **rpm** | **CO_2_ %** | **Inc. time (hr)** | **Mating vol^¤^ (mL)** | **Plated vol* (mL)** | **Bacterial pellet^§^** | **TC^#^** |
| --- | --- | --- | --- | --- | --- | --- | --- | --- | --- |
| M | Mb pI100^P^ (9) | Mb p643^G^ (9) | 150 | 0 | 24 | 1 | 0.9 | Yes | Present |
| N | Mb pI100^P^ (9) | Mb p643^G^ (9) | 150 | 0 | 24 | 2 | 0.9 | No | Absent |
| Ñ | Mb pI100^P^ (9) | Mb p643^G^ (9) | 150 | 5 | 24 | 2 | 0.9 | No | Absent |
| O | Mb pI100^P^ (9) | Mb p643^G^ (9) | 100 | 5 | 5, 10, 15, 20 | 2 | 0.9 | No | Present |
| P | Mb pI100^P^ (9) | Mb p643^G^ (9) | 100 | 5 | 2, 4, 6, 8 | 2 | 0.9 | No | Present |
| S | Mb pI100^P^ (9) | Mb p643^G^ (9) | 100 | 5 | 1, 2, 4 | 2 | 0.9 | No | Present |

^¤^Fresh liquid media volume used to resuspend the bacterial pellet originated after spinning down 2 mL worth of parent A+B mixed culture.

*Conjugation broth volume plated per incubation time onto SP4 agar supplemented with antimicrobials for the detection of transconjugants.

^§^Bacterial pellet present at the bottom of the tube/ flask after the mating incubation period was over.

^#^Transconjugants,

# 9. Approaches tested for the detection of conjugation in *Mycoplasmopsis bovis*

Approaches initially followed to identify conjugation candidates:

- From MJC draft genomes, isolates containing the CDSs thought to be essential for conjugation (11) and
- The detection of cMICE by PCR (15).

Initial conjugation experiments following the *reference* method provided none or very low transconjugants (**Supp. Table S13**). Therefore, different approaches and protocol deviations were tested to elucidate whether *M. bovis* still preserved its conjugation capabilities through MICE.

*Conjugation *reference* conditions (1): SP4 media with ampicillin ( to avoid media contamination), overnight incubation at 37 °C, no CO_2_ supplementation, no shaking, 1:1 initial parent’s proportions.

- Different incubation times of the conjugation broth i.e., 3 hr, 6 hr, 12 hr, 15 hr, 18 hr, 36 hr, 48 hr. However, this did not result in successful conjugation.
- Different initial proportions of the parent strains i.e. 1:1, 1:10, and 1:100 (16). However, this did not result in successful conjugation.
- Self-conjugation to test the functionality of the MICE conjugation machinery.

Self-conjugation between strains of the same isolate tagged with different ARGs has been reported for *M. agalactiae* (15). Therefore, this was tested in some of our *M. bovis* field isolates that were cMICE-positive to rule out defective MICE machinery and the absence of conjugation due to a possible incompatibility between different isolates i.e. the presence of a non-compatible recipient. However, this did not result in successful conjugation.

- A different media preparation protocol.

It has been reported that sterilizing agar separately from the other media components, and reducing the agar concentration have the potential to improve microorganisms growth (10.1128/spectrum.03161-22). Additionally, the addition of extra water before the sterilization process to account for the water loss keeps Maillard reaction products (MRP) at lower levels. MRP have the potential to interfere with microbial growth and other physiological processes (17, 18). Theoretically, the use of a microwave, instead of an autoclave, would represent a better sterilization option to keep MRP levels to a minimum. However, all the different microwaving times and potency tested provided high rates of media contamination (data not shown). Therefore, the microwave was discarded and plates were prepared sterilizing the agar separately. However, this did not result in successful conjugation.

- The use of a media different to SP4.

In *Streptococcus thermophilus*, ICE excision was detected in two different rich media i.e., LM17 and HJGL, but conjugation was only reported in LM17 and not in HJGL (19). This highlights the limited knowledge we have about *in vitro* bacterial conjugation. Therefore, aside from SP4, other media that supported the growth of *M. bovis* was tested for conjugation i.e., PPLO and Eaton’s under the *reference* conjugation conditions. However, this did not result in successful conjugation.

- Mating pairs between 2 different isolates that were cMICE-positive instead of positive x negative.

A deficient MICE-CDS14 can be trans-complemented by functional CDS14 present in co-resident MICE from the same chromosome, or from neighbouring cells (16). Moreover, *M. agalactiae* 5632 was able to acquire a 4^th^ MICE copy by conjugation (15) suggesting the absence of exclusion factors that would prevent the entrance of a new MICE copy when similar ones already exist within a chromosome. Therefore, in the event that conjugation was not observed due to the presence of deficient MICE-CDSs in our isolates, different *M. bovis* isolates that were cMICE-positive were mated (i.e. 643, C297A, 646). However, this did not result in successful conjugation.

- Screening for the presence of more than one cMICE within the same *M. bovis* chromosome.

*Mycoplasmopsis agalactiae* 5632 has been extensively used in conjugation experiments. It contains 3 almost identical copies of ICEA capable of self-excision and circularization (20). The interaction between different co-resident MICE has been described as complex (16). And even though it seems that only one functional MICE copy is needed for conjugation to happen (*M. agalactiae* 4867 (21)), a subset of isolates (n = 25) was screened for the presence of more than one cMICE in the event this could aid in conjugation.

For this, the left1/right2 cMICE primers were used (1) and the cPCR products were sent for Sanger sequencing (Eurofins Genomics). Within the PCR amplicons, the 6 nt MICE coupling region was identified and determined whether it presented a clear nt sequence (indicative of the presence of a single) or not (indirect evidence of the presence of more than 1 cMICE) (data not shown). However, this did not result in successful conjugation.

- Increase the conjugation volume plated on SP4.

According to the mycoplasma conjugation *reference* protocol (1), 1 mL of mating broth is set per conjugation experiment and 3 x 0.3 mL of that broth are plated onto SP4 agar supplemented selective antimicrobials. In our initial conjugation experiments, only 1 and 2 TC colonies were obtained from two different mating pairs prompting the question whether the conjugation frequency was that low that could not be detected by plating 0.9 mL of the conjugation broth. Therefore, that volume was increased to 4 mL. However, this did not result in successful conjugation.

- Mating TF pools with individual TF clones.

Since *M. bovis* field isolate 643 was the common isolate present in both successful conjugation experiments following the *reference* method, individual 643-GEN TF clones were used for conjugation with TF pools of 057 and I100 (**Supp. Table S13**). This strategy was followed under the assumption that only one or a few 643-TFs were able to conjugate since the ARGs from the pMT85 plasmids are inserted in the mycoplasma chromosome at random (16). This at random insertion has the potential to disrupt relevant CDSs including MICE CDSs that could potentially affect the conjugation capabilities of some 643-TF clones. However, this did not result in successful conjugation.

- Shaking the conjugation broth.

Shaking bacterial cultures increased *E. coli* conjugation frequencies up to 4 times (22). Even though *reference* mycoplasma conjugation protocol specify to incubate the mating broth without shaking (1), conjugation was tested on an orbital shaker under different conditions (**Supp. Table 14**). This change initially provided consistent conjugation events between *M. bovis* 643 x I100.

# 10. Optimized *Mycoplasmopsis bovis* conjugation protocol

Our optimized conjugation protocol for *M. bovis* is comparable to the previously published one (1), with the main difference being the mating broth incubation conditions i.e., under agitation and 5% CO_2_ (instead of static and no CO_2_).

***Day 1***

- Inoculate 1 mL of fresh SP4 broth (screw-cap 1.5 mL tube) with 10 µL of *M. bovis* -70 °C culture stock^1^ at ~10^9^ CFU/mL.
- If a pool of transformants is used per mating parent, culture each transformant individually.
- Incubate at 37 °C, 5% CO_2_, and no shaking for approximately 24 h^2^ making sure the screw-caps are slightly loose.

***Day 2***

- If a pool of transformants is used per mating parent, pool all the transformants per mating parent in a 50 mL tube and mix gently.
- In a 2 mL sterile microcentrifuge tube, mix 1 mL of each conjugation (transformants pool) parent culture^3^.
- Centrifuge at 8,000 x g, 5 min, and room temperature (20-25 °C).
- Discard supernatant.
- Carefully, resuspend the bacterial pellet in 1 mL of fresh SP4 broth (pre-incubated in the CO_2_ incubator) and transfer 2 x 1 mL of bacteria to a 25-50 mL sterile Erlenmeyer flask (loose lid).
- Incubate up to 8 h, at 37 °C, 5% CO_2_^4^, and orbital shaking at 100 rpm^5^ (this growth represents the mating mix).
- Spread plate 2 x 450 µL of the mating mix onto SP4 agar (gridded square petri dish, 100 x 100 mm) supplemented with the appropriate antibiotics for the selection of transconjugants.
- Incubate the plates for up to 5 days, at 37 °C, and no CO_2_^6^. Transconjugants normally can be visible after 2 days of incubation.
- To determine CFU/mL of the mating mix, perform 10-fold serial dilutions in 90 µL of DBPS 1X + FBS 5% v/v, up to dilution 10^-7^. Each dilution is spot-platted (10 µL) in triplicates on SP4 agar supplemented with the appropriate antibiotics^7^ (gridded square petri dish, 100 x 100 mm, 1 spot per square).
- Incubate the enumeration plates at 37 °C.

***Days 4-7***

- Check the conjugation plates for the presence of colonies under a stereoscopic microscope. If present, 3 cloning or subculturing steps are performed in SP4 media supplemented with the appropriate antibiotics. The last broth culture is filtered through a 0.22 µm pore filter before further analyses and long term storage.
- Check the enumeration plates for the presence of colonies under a stereoscopic microscope. If present, count them when fully developed and determine conjugation efficiency as previously described (1).

***Verification of transconjugants***

A. Direct qPCR method: follow the protocol specified in the main manuscript’s Material and Methods section using the primers that amplify the resistance genes present in the conjugation parent strains (**Supp. Table S12**).

B. Genomic DNA purification + conventional PCR method: centrifuge 1.5-2 mL of 24-48 h transconjugant growth (8,000 x g, 10 min, 4 °C), perform a gDNA extraction (*DNeasy Blood & Tissue* DNA isolation kit; Qiagen), and amplify the resistance genes present in the conjugation parent strains by conventional PCR (**Supp. Table S12**).

***Notes***

^1^*M. bovis* -70 °C culture stocks: when broth containing an animal serum was used to grow mycoplasmas, there is no need to add a cryopreservative like glycerol to ensure cell viability (CLSI document M43-A). Moreover, 30% glycerol was toxic to some mycoplasma species like *M. arginini* (23).

^2^Under 5% CO_2_, the SP4 media experienced a color change from red/pink to orange/yellow regardless of *M. bovis* presence (it contains phenol red as a pH indicator). However, when *M. bovis* growth was present, the color change was stronger than for only-media and turbidity could be observed.

^3^If a pool of transformants is used as a conjugation parent, use 1 mL of the pool containing equal proportion of each transformant. The initial proportion between conjugation parents should be close to 1:1; this proportion could be modified if the growth rates between conjugation parents is substantially different.

^4^5% of CO_2_ was required during conjugation because the culture was carried out under agitation, to ensure optimum growing conditions.

^5^We used a Heidolph Unimax 1010 orbital shaker with an orbit diameter of 20 mm.

^6^The incubation of SP4 plates supplemented with antimicrobials under a 5% CO_2_ atmosphere promotes the growth of non-transconjugants.

^7^Conjugation efficiency can be calculated as the ratio of transconjugants/ total CFU in the mating mix, for which serial dilutions are spot-plated onto SP4 agar with no antibiotics. But if the conjugation efficiency is expressed by transconjugants per donor or recipient, then the appropriate antimicrobials are supplemented to SP4 agar (1).

**Supplementary Figure S5.** Representation of the optimized conjugation protocol for *Mycoplasmopsis bovis*


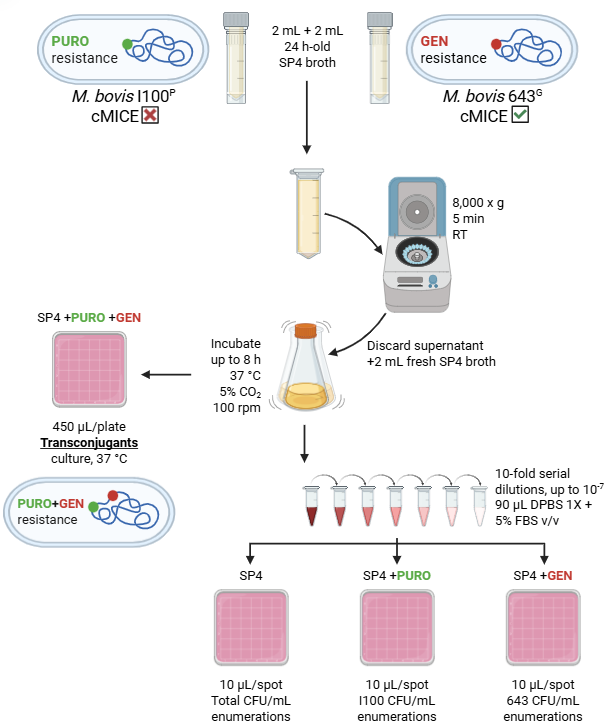


CFU, colony forming unit; cMICE, circular form of mycoplasma integrative and conjugative elements; GEN, gentamicin; PURO, puromycin; RT, room temperature.

# 11. Supplementary references

1. Sagne E, Citti C, Dordet-Frisoni E. Bacterial conjugation protocol for ruminant mycoplasmas. Bio Protoc. 2021;11(2):e3893.

2. Jelinski M, Kinnear A, Gesy K, Andres-Lasheras S, Zaheer R, Weese S, et al. Antimicrobial sensitivity testing of *Mycoplasma bovis* isolates derived from western Canadian feedlot cattle. Microorganisms. 2020;8(1):124.

3. Nicholas R, Ayling R, McAuliffe L. Mycoplasma diseases of ruminants. United Kingdom: CAB International; 2008.

4. Freundt EA. Methods in Mycoplasmology: Academic Press; 1983 1983.

5. Gutgemann F, Muller A, Churin Y, Kumm F, Braun AS, Yue M, et al. Toward a method for harmonized susceptibility testing of *Mycoplasma bovis* by broth microdilution. J Clin Microbiol. 2023;61(8):e0190522.

6. Ayling RD, Rosales RS, Barden G, Gosney FL. Changes in antimicrobial susceptibility of *Mycoplasma bovis* isolates from Great Britain. Vet Rec. 2014;175(19):486.

7. Andrés-Lasheras S, Ha R, Zaheer R, Lee C, Booker CW, Dorin C, et al. Prevalence and risk factors associated with antimicrobial resistance in bacteria related to bovine respiratory disease—A broad cross-sectional study of beef cattle at entry Into canadian feedlots. Front Vet Sci. 2021;8:1.

8. Ambroset C, Peticca A, Tricot A, Tardy F. Genomic features of *Mycoplasma bovis* subtypes currently circulating in France. BMC Genomics. 2022;23(1):603.

9. Garcia-Galan A, Baranowski E, Hygonenq MC, Walch M, Croville G, Citti C, et al. Genome mosaicism in field strains of *Mycoplasma bovis* as footprints of in-host horizontal chromosomal transfer. Appl Environ Microbiol. 2022;88(1):e0166121.

10. Triebel S, Sachse K, Weber M, Heller M, Diezel C, Holzer M, et al. De novo genome assembly resolving repetitive structures enables genomic analysis of 35 European *Mycoplasmopsis bovis* strains. BMC Genomics. 2023;24(1):548.

11. Citti C, Dordet-Frisoni E, Nouvel LX, Kuo CH, Baranowski E. Horizontal gene transfers in Mycoplasmas (Mollicutes). Curr Issues Mol Biol. 2018;29:3-22.

12. Andres-Lasheras S, Zaheer R, Ha R, Lee C, Jelinski M, McAllister TA. A direct qPCR screening approach to improve the efficiency of *Mycoplasma bovis* isolation in the frame of a broad surveillance study. J Microbiol Methods. 2020;169:105805.

13. Sansevere EA, Luo X, Park JY, Yoon S, Seo KS, Robinson DA. Transposase-mediated excision, conjugative transfer, and diversity of ICE6013 elements in *Staphylococcus aureus*. J Bacteriol. 2017;199(8):e00629-16.

14. Tardy F, Mick V, Dordet-Frisoni E, Marenda MS, Sirand-Pugnet P, Blanchard A, et al. Integrative conjugative elements are widespread in field isolates of Mycoplasma species pathogenic for ruminants. Appl Environ Microbiol. 2015;81(5):1634-43.

15. Dordet Frisoni E, Marenda MS, Sagne E, Nouvel LX, Guerillot R, Glaser P, et al. ICEA of *Mycoplasma agalactiae*: a new family of self-transmissible integrative elements that confers conjugative properties to the recipient strain. Mol Microbiol. 2013;89(6):1226-39.

16. Baranowski E, Dordet-Frisoni E, Sagne E, Hygonenq MC, Pretre G, Claverol S, et al. The integrative conjugative element (ICE) of *Mycoplasma agalactiae*: key elements involved in horizontal dissemination and influence of coresident ICEs. mBio. 2018;9(4):e00873-18.

17. Bazana LCG, Carvalho AR, Mace M, Fuentefria AM. The influence of the microwave oven on the production of solid culture medium and quality of microbial growth. An Acad Bras Cienc. 2022;94(3):e20211104.

18. Bhattacharjee MK, Delsol JK. Does microwave sterilization of growth media involve any non-thermal effect? J Microbiol Methods. 2014;96:70-2.

19. Carraro N, Libante V, Morel C, Decaris B, Charron-Bourgoin F, Leblond P, et al. Differential regulation of two closely related integrative and conjugative elements from *Streptococcus thermophilus*. BMC Microbiol. 2011;11:238.

20. Marenda M, Barbe V, Gourgues G, Mangenot S, Sagne E, Citti C. A new integrative conjugative element occurs in *Mycoplasma agalactiae* as chromosomal and free circular forms. J Bacteriol. 2006;188(11):4137-41.

21. Dordet-Frisoni E, Faucher M, Sagne E, Baranowski E, Tardy F, Nouvel LX, et al. Mycoplasma chromosomal transfer: a distributive, conjugative process creating an infinite variety of mosaic genomes. Front Microbiol. 2019;10:2441.

22. Headd B, Bradford SA. Physicochemical factors that favor conjugation of an antibiotic resistant plasmid in non-growing bacterial cultures in the absence and presence of antibiotics. Front Microbiol. 2018;9:2122.

23. Dabrazhynetskaya A, Furtak V, Volokhov D, Beck B, Chizhikov V. Preparation of reference stocks suitable for evaluation of alternative NAT-based mycoplasma detection methods. J Appl Microbiol. 2014;116(1):100-8.
